# Reducing OGT and *O*-GlcNAcylation enhance the anticancer effects of oxaliplatin in SW620 metastatic colorectal cancer cells

**DOI:** 10.1371/journal.pone.0341971

**Published:** 2026-02-10

**Authors:** Thirasak Bunsuk, Juthamard Chantaraamporn, Photsathorn Mutapat, Penchatr Diskul Na Ayudthaya, Daranee Chokchaichamnankit, Chantragan Srisomsap, Jisnuson Svasti, Voraratt Champattanachai

**Affiliations:** 1 Applied Biological Sciences: Environmental Health Program, Chulabhorn Graduate Institute, Bangkok, Thailand; 2 Laboratory of Biochemistry, Chulabhorn Research Institute, Bangkok, Thailand; University of Rochester, UNITED STATES OF AMERICA

## Abstract

*O*-GlcNAcylation, a single attachment of N-acetylglucosamine (GlcNAc) on serine/threonine residues of nuclear-cytoplasmic proteins, is frequently upregulated in various cancers and implicated in several aspects of tumor progression. Growing evidence reports that treatments of chemotherapeutic drugs may activate protein *O*-GlcNAcylation. However, its precise role in modulating chemotherapeutic responses, particularly in colorectal cancer (CRC), remains poorly defined. Herein, we investigate the biological effects of oxaliplatin (OXA), a first-line chemotherapy drug for patients with metastatic CRC, and protein *O*-GlcNAcylation reduction in SW620 metastatic CRC cells. OXA treatment alone reduced cell viability as well as proliferation, and increased the levels of protein *O*-GlcNAcylation and GFPT1, the rate limiting enzyme of hexosamine biosynthetic pathway which is a nutrient sensor of glucose metabolism. Inhibition of protein *O*-GlcNAcylation via genetic knockdown of *O*-GlcNAc transferase (OGT) or chemical inhibition (OSMI-1) markedly enhanced SW620 sensitive to OXA. This was evidenced by decreased cell viability and proliferation, increased cell apoptosis, and cell cycle arrest. Mass spectrometry-based proteomics and bioinformatics analysis revealed that the combination of OGT knockdown and OXA treatment majorly downregulated several ribosomal proteins. In addition, OXA treatment and OGT knockdown altered proteins involved in critical pathways including DNA synthesome complex, glycolytic process, negative regulation of gene expression, cell cycle process, and negative regulation of protein phosphorylation. Specifically, OGT downregulated several ribosomal proteins, and OGT knockdown influenced proteins across the identified pathways. Taken together, these findings demonstrate that reducing OGT and protein *O*-GlcNAcylation may enhance the sensitivity of CRC cells to OXA, and the altered pathways may offer new insights into potential mechanisms for overcoming CRC chemoresistance.

## 1. Introduction

Colorectal cancer (CRC) is a common cancer that primarily affects the elderly, with most cases occurring in people over 50 years of age [1]. Based on the 2024 report on the global cancer statistics, CRC is the third rank of diagnosed cancers, following lung and female breast cancer, and is also the second rank leading cause of cancer-related deaths, after lung cancer [2]. CRC development takes over a decade and is driven by the accumulation of numerous mutations in cells in intestinal tissues, resulting in uncontrollable cell expansion and transformation into a malignant tumor. Once cancer cells are formed at the primary site, they have ability to invade to neighboring cells and metastasize to distant (secondary) sites such as the liver and brain [3].

Metabolic reprograming has been extensively explored in the context of oncogenesis and is one vital characteristic cancer hallmark [4]. Changes in glucose metabolism in cancer affect several metabolic pathways, including the hexosamine biosynthesis pathway (HBP) [5]. The end-product of HBP is N-acetylglucosamine (GlcNAc) which is a substrate for both classical glycosylation and *O*-GlcNAcylation of proteins. Unlike asparagine N-GlcNAylation, a classical N-linked attachment of dolichol-GlcNAC_2_Man_9_Glc_3_ on asparagine by oligosaccharyltransferase (OST) that occurs in the endoplasmic reticulum, *O*-GlcNAcylation involves the attachment of a single GlcNAc molecule to serine and threonine residues of cytoplasmic and nuclear proteins [6]. This modification is dynamically regulated by two key enzymes; *O-*GlcNAc transferase (OGT) and *O-*GlcNAcase (OGA), which are responsible for adding and removing sugar donors, respectively and may interplay with phosphorylation [7]. Previously, our group and others reported that the levels of OGT and protein *O*-GlcNAcylation were upregulated in CRC tissues compared to those of normal tissues [8,9]. In addition, our group also showed that the level of protein *O*-GlcNAcylation in the CRC metastatic clone (SW620) was higher than that of the CRC isogenic primary clone (SW480) [10]. We also demonstrated that OGT knockdown inhibited colony formation of those CRC cell lines, especially in the metastatic clone. These data suggest that protein *O*-GlcNAcylation and OGT may play important roles in cancer progression, especially in the metastatic CRC.

Chemotherapy remains a standard treatment for the metastatic and advanced stages of cancers. Several lines of evidence demonstrate that cellular stresses induced by chemotherapy enhance the global protein *O-*GlcNAcylation and this augmentation is thought to activate signaling pathways that promote cell survival and protection [11–13]. Cisplatin enhances protein *O*-GlcNAcylation in non-small cell lung cancer cells [11]. Doxorubicin increased *O*-GlcNAcylation level and suppression of this modification reduced the resistance of breast cancer cells to chemotherapy [12]. In colon cancer, inhibition of protein *O*-GlcNAcylation changes the cellular response from protective senescence to therapeutic apoptosis [14]. These findings collectively highlight the critical role of protein *O*-GlcNAcylation in regulating cancer chemoresistance.

Oxaliplatin (OXA) is a commonly used chemotherapy drug approved for the treatment of advanced CRC [15]. Its cytotoxicity is thought to inhibit DNA synthesis by formation of platinum-DNA adducts, thus inducing cellular stress and causing cell death. In this study, as OXA treatment targets to metastatic cancer, and our previous report indicates that *O*-GlcNAcylation is up-regulated in the metastatic CRC, SW620, we decide to use this cell line to investigate whether the reduction of protein *O*-GlcNAcylation level by OSMI-1, an OGT inhibitor, or OGT knockdown affected cell responses in the combination with OXA treatment. In addition, the biological effects of OGT knockdown with oxaliplatin were investigated. Lastly, mass spectrometry-based proteomics and bioinformatics analysis were applied in order to find the potential signaling pathways upon treatments by OXA and OGT reduction.

## 2. Materials and methods

### 2.1. Cell cultures

CRC cell lines including SW480 and SW620 were purchased from the American Type Culture Collection (ATCC; Rockville, MD, USA). They were cultured in Roswell Park Memorial Institute (RPMI) 1640 (Thermo Fisher Scientific, Waltham, MA, USA) containing 10% Fetal bovine serum (HyClone, GE Healthcare Life Sciences, Logan, UT, USA), and 1%Antibiotic-Antimycotic (100 units/mL of penicillin, 1000 μg/mL of streptomycin, and 0.25 μg/mL of Fungizone® Antimycotic, Anti-Anti, Gibco). Cells were cultivated in cell culture flasks and maintained in 5% CO_2_ humidity incubator at 37°C. Cells were subcultured once it reaches 70–90% confluence by trypsinization.

### 2.2. Treatments of OXA and OSMI-1

OXA and OSMI-1 (an inhibitor of OGT) were purchased from MedChemExpress (Monmouth Junction, NJ, USA) and resuspended in dimethyl sulfoxide (DMSO). SW620 cells were seeded in appropriate well sizes (i.e., 5,000 cells/well for 96-well plates and 200,000 cells/well for 6-well plates) for 24 hours, then treated with OXA for additional 48 hours. For OSMI-1 treatment, this was pretreated at 50 µM for 1 hour prior to treatment with OXA. Treatment of OSMI-1 at various doses was performed (S2 Fig in S1 Data). Control cells in all experiments were treated with 0.2% DMSO as vehicle.

### 2.3. Cell viability assay

Cell viability was performed using CellTiter 96® AQ_ueous_ One Solution Cell Proliferation Assay (MTS) of Promega Corporation (Madison, WI, USA) as previously described with some modifications [10]. Briefly, at the end of treatments, the cells in wells of 96-well plates were incubated with MTS solution for 2 hours and then the signals were determined by a 96-well plate reader (Molecular Devices, San Jose, CA, USA). Results are expressed as a percentage relative to the untreated control. Each experiment was performed in at least 3 independent replicates and 3 technical replicates for each condition.

### 2.4. Cell proliferation assay

Cell proliferation was performed using BrdU Cell Proliferation ELISA Kit (colorimetric) of Abcam (AB126556; Abcam, Cambridge, UK) according to the manufacturer’s instructions. Briefly, the cells in wells of 96-well plates were incubated with BrdU solution for 24 hours prior the end point of treatment and then the signals were determined by a 96-well plate reader (Molecular Devices, San Jose, CA, USA) at a dual wavelength of 450/550 nm. Results are expressed as a percentage relative to the untreated control. Each experiment was performed in at least 3 independent replicates and 3 technical replicates for each condition.

### 2.5. Flow cytometric analysis of apoptosis and cell cycle distribution

The apoptotic profiles and cell cycle analysis of SW620 cells treated with OXA and *O*-GlcNAc treatments were assessed by Muse® Cell Analyzer (EMD Millipore, Hayward, CA, USA) using Muse® Annexin V & Dead Cell Kit (Cytek Biosciences, San Diego, CA, USA) or Muse® Cell Cycle kit (Cytek Biosciences, San Diego, CA, USA), according to the manufacturer’s instructions. Then, the cells were subjected to apoptotic detection or cell cycle analysis using Muse^TM^ Cell Analyzer (EMD Millipore Corporation, Hayward, CA, USA). Results are expressed as a percentage relative to the untreated control of total apoptosis (7-AAD (-)/(+) and Annexin V (+)) or proportion of the cells in each phase of cell cycle (G0/G1, S and G2/M), respectively. Each experiment was performed in at least 3 independent replicates.

### 2.6. Immunoblot analysis

Immunoblotting was performed as previously described with some modifications [16]. Briefly, cells were harvested and lyzed in RIPA buffer containing 1% protease inhibitor cocktail (Sigma‑Aldrich) and 10 μM TMG (an OGA inhibitor, MedChemExpress). Protein concentrations were measured using a Bio‑Rad Protein assay (Bio‑Rad Laboratories, Hercules, CA, USA). Protein samples (20 μg) were loaded on a 10% SDS‑gel, resolved using SDS‑PAGE and transferred to PVDF membranes (EMD Millipore Corporation). The levels of O‑GlcNAcylation, OGT, OGA, GFPT1 and β‑actin were determined using immunoblotting with primary antibodies, including antibodies against *O*‑GlcNAc‑modified proteins RL2 (1:1,000; AB2739; Abcam, Cambridge, UK), OGT (1:1,000; AB177941, Abcam), OGA (1:10,000; AB124807, Abcam), GFPT1 (1:1,000; AB125063) and β‑actin (1:20,000; mAb3700; Cell Signaling Technology, Danvers, MA, USA), with overnight incubation at 4˚C in 1% PBS‑casein blocking buffer (Bio‑Rad Laboratories) as previously described [17]. The membranes were incubated with the corresponding secondary antibodies, including swine anti‑rabbit (1:5,000; P‑0217; Dako; Agilent Technologies, Santa Clara, CA, USA) or rabbit anti‑mouse immunoglobulins (1:5,000; P‑0260; Dako; Agilent Technologies). Immunoblots were developed by SuperSignal West Pico Plus Chemiluminescent Substrate (Thermo Fisher Scientific) and protein signals were detected by ImageQuant LAS4000 (GE Healthcare). Each experiment was performed in at least 3 independent replicates.

### 2.7. OGT knockdown

OGT knockdown was performed using Stealth RNAi technology as previously described with some modifications [16]. Stealth RNAi oligonucleotides against OGT (siOGT) and scrambled negative control medium GC duplex (siSC; cat. no. 12935300), were purchased from Invitrogen (Thermo Fisher Scientific, Inc.). The sense and anti-sense sequences of siOGT were 5’‑ GGG UUA CCA GAA GAU GCC AUC GUA U-3’ and 5’-AUA CGA UGG CAU CUU CUG GUA ACC C-3’, respectively. The cells in wells of 6-well plates were transfected with 20 nM stealth siOGT or siSC using Lipofectamine® 3000 reagent (Invitrogen; Thermo Fisher Scientific) and performed according to the manufacturer’s instructions. After 48 hours of transfection, cells were re-seeded in appropriate wells and treated with OXA for an additional 48 hours to measure cell viability, apoptosis, and cell cycle distribution as mentioned above.

### 2.8. Sample preparation for mass spectrometer

Protein samples were obtained from four groups including (1) siSC, (2) siSC with 10 µM OXA, (3) siOGT, and (4) siOGT with 10 µM OXA in three biological replicates per group (total samples = 12). At the end of treatments, cells were harvested as cell pellets and stored at −80°C. The frozen packed cells were lyzed in lysis buffer (8M urea, 75 mM NaCl and 50 mM Tris, pH 8.2) containing 1% protease inhibitor cocktail (Sigma‑Aldrich). Protein concentration was measured using a Bio‑Rad Protein assay (Bio‑Rad Laboratories). A total of 25 μg of each sample was reduced with 5 mM dithiothreitol (DTT) at 56˚C for 30 min, followed by an alkylation step with 15 mM iodoacetamide (IAM) in the dark at room temperature for 30 min. Subsequently, samples were digested in solution with 1 μg Trypsin/LysC Mix (Promega). The samples were incubated at 37°C for 4 hours for the LysC digestion (8M urea), and then added with 50 mM Tris buffer (pH 8.2) to reduce the urea concentration (<1M urea) and further incubated at 37°C, overnight (14 hours). After digestion, samples were acidified with formic acid and then desalted using Pierce® C18 spin columns (Thermo Fisher Scientific), and dried by a CentiVap Vacuum Concentrator (Labconco, Kansas City, Mo, USA) and kept at -80^o^C until use.

### 2.9. Liquid chromatography tandem mass spectrometry analysis

Liquid chromatography coupled to high-resolution trapped ion mobility spectrometry tandem mass spectrometry (LC-TIMS-MS/MS) was performed by a nanoflow liquid chromatography system (Thermo Fisher Scientific) coupled online to timsTOF Pro, a TIMS quadrupole time-of-flight mass spectrometer (Bruker Dal-tonics) as previously described [18]. Dried peptides were resuspened in 0.1% formic acid and then measured using a Pierce Quantitative Colorimetric Peptide assay (Thermo Fisher Scientific). Two hundred nanograms of peptides were loaded on a capillary C18 column (25 cm x 75 μm, 1.6 μm and 120 Å pore size; IonOpticks, Fitzroy, VIC, AUS). Peptides were separated at 50°C using a 55 min gradient at a flow rate of 300 nL/min (solution A: 0.1% formic acid in 100% water; solution B: 0.1% formic acid in 100% acetonitrile). A stepwise gradient of 2% to 40% B was applied for 45 min and finished with a wash at 85% B for an additional 10 min. The timsTOF Pro was operated in data-dependent (ddaPASEF) mode. Data-dependent acquisition was performed using one MS1 survey TIMS-MS and ten PASEF MS/MS scans per acquisition cycle with a near 100% duty cycle. Ion mobility resolution was set to 1/K0 = 1.6 Vs cm-2 to 0.6 Vs cm-2 over a ramp time in the dual TIMS analyzer of 100 ms. Singly charged precursor ions were excluded with a polygon filter. An active exclusion time of 40-second elution was applied to precursors that reached 20,000 intensity units. The collisional energy was ramped stepwise as a function of ion mobility at 1/K0 = 1.6 Vs cm-2–1/K0 = 0.6 Vs cm-2.

### 2.10. Protein identification and quantification

Raw MS data were analyzed by PEAKS Studio (Bioinformatics Solutions, Waterloo, ON, Canada). All MS spectral data obtained from three biological replicates of each sample group were used (total samples = 12). The data were searched against the SwissProt database of Homo sapiens (downloaded 2023/11/24). The following parameters were specified: digested by trypsin, with one missed cleavage; precursor mass tolerance was 10 ppm; fragment mass tolerance: 0.01 Da, minimum charge: 2, maximum charge: 5, carbamidomethylation of cysteines as a fixed modification, methionine oxidation and acetylation N-terminal as variable modifications. The false discovery rate (FDR) estimation through a decoy database was set to 1%. An FDR of 1% with one minimum unique peptide per protein were used as a filter for identification. Contaminant proteins were removed prior to determine the relative protein abundance among sample groups.

Protein quantification from all samples (n = 12) were log_2_-transformed, normalized and imputed for missing values as described in Aguilan et al. 2020 [19]. For identifying the significantly different protein expression among the sample groups, one-way analysis of variance (ANOVA) with Tukey’s multiple comparison test were performed using the Real Statics v9.0 on Microsoft Excel 365 for Windows (Real Statistics Resource Pack Software, Copyright 2013–2025). The results of one-way ANOVA were evaluated at significance level of P < 0.05.

### 2.11. Bioinformatics analysis

The differentially expressed proteins with one-way ANOVA P < 0.05 were considered. The principal component analysis (PCA) function prcomp and hierarchical clustering function hclust in the R language were used to analyze high-dimensional data and perform unsupervised clustering of significant differential expression of proteins among the treated and untreated groups of SW620 cells. The biological processes of proteins in each cluster were analyzed by Metascape, v3.5.20240901 [https://metascape.org] [20]. Then, the enriched ontology clusters were visualized using function ggplot2 and GOChord.

### 2.12. Statistical analysis

All data are presents as the mean ± standard deviation of at least three biological replicates. One-way ANOVA was used, followed by Tukey’s multiple comparison test using Prism v.10 (GraphPad Software, San Diego, CA). Statistically significant differences between groups were deﬁned as P < 0.05.

## 3. Results

### 3.1. *O*-GlcNAcylation and OXA treatment in CRC cells

**Fig 1:**
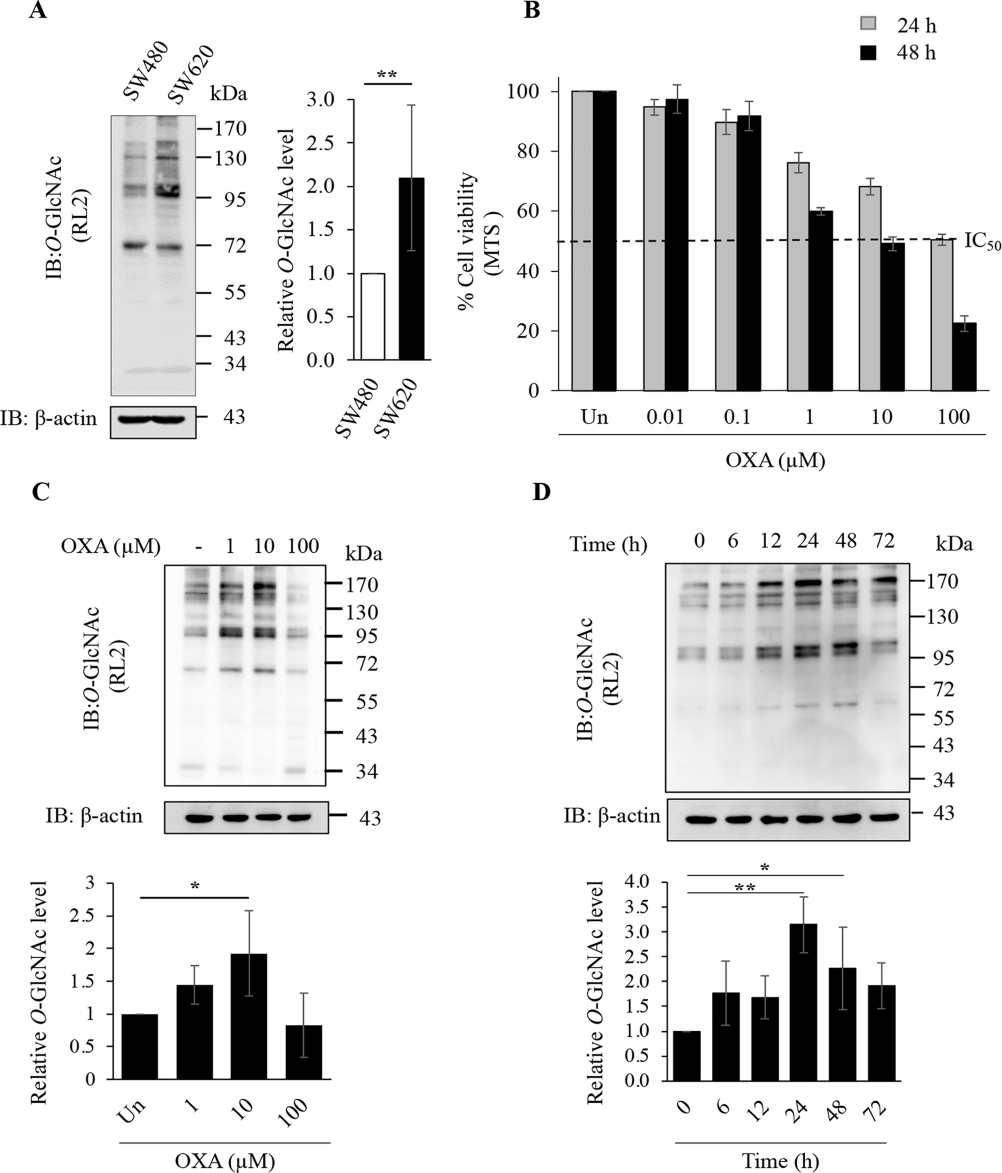
*O*-GlcNAcylation level and OXA treatment in CRC cells. (A) Representative immunoblots (IB) of *O*-GlcNAcylation (RL2) and β-actin and its relative *O*-GlcNAcylation levels of SW480 and SW620 CRC cells (6 independent replicates). (B) Bar graph represents the cell viability of SW620 cells treated with OXA. Cells were treated with OXA at various concentrations for 24 (grey) and 48 (black) hours and assessed by the MTS assay (3 independent replicates). IC_50_ means the half maximal inhibitory concentration. (C) and (D) Representative immunoblots of *O*-GlcNAcylation (RL2) and β-actin of SW620 cells treated with OXA and its relative *O*-GlcNAcylation levels by various dose at 48 hours (4 independent replicates) and various time at 10 µM (3 independent replicates), respectively. *O*-GlcNAcylation level was determined by immunoblotting as described in the method. β-actin was used as a protein loading control. Data are presented as the relative percentage normalized by the untreated control, and values are expressed as the mean ± standard deviation. *P < 0.05 and **P < 0.01.

The *O*-GlcNAcylation level was determined in two isogenic CRC cell lines including SW480 (non-metastatic clone) and SW620 (metastatic clone) by immunoblotting. The results revealed that *O*-GlcNAcylation level in SW620 cells was significantly higher than that of SW480 cells (Fig 1A). This suggests that *O*-GlcNAcylation may correlate to the metastatic phenotype. In addition, we found that *O*-GlcNAcylation level was increased by the treatment of OXA and 5-FU in both metastatic SW620 and non-metastatic SW480 CRC cells, but they were different in degree of action and time of treatment (S1 Fig in S1 Data). This finding indicates that augmentation of *O*-GlcNAcylation may also corelate with chemotherapy drug treatments. Based on our preliminary data, SW620 cells treated with OXA showed the highest level of *O*-GlcNAcylation augmentation, thus they were chosen to further experiments. SW620 cells were treated with various concentrations of OXA for 24 and 48 hours. The results showed a dose-dependent decrease in cell viability for both treatment durations with its IC₅₀ about 100 µM and 10 µM at 24- and 48-hours of treatments, respectively (Fig 1B). OXA treatment resulted in a significant increase in *O*-GlcNAcylation level at 10 µM, while it was not significantly different at 1 µM but tended to be increased when compared to that of the untreated cells (Fig 1C). In addition, OXA treatment led to a significant increase of *O*-GlcNAcylation level at 24 and 48 hours (Fig 1D). This data indicates that an increase in *O*-GlcNAcylation level in SW620 cells treated with OXA may act as a cellular stress response. According to our data, the OXA at 10 µM for 48 hours of treatment was chosen in the following experiments to observe the effects of alteration of OGT and *O*-GlcNAcylation.

### 3.2. Effects of OXA and OSMI-1 treatments

**Fig 2:**
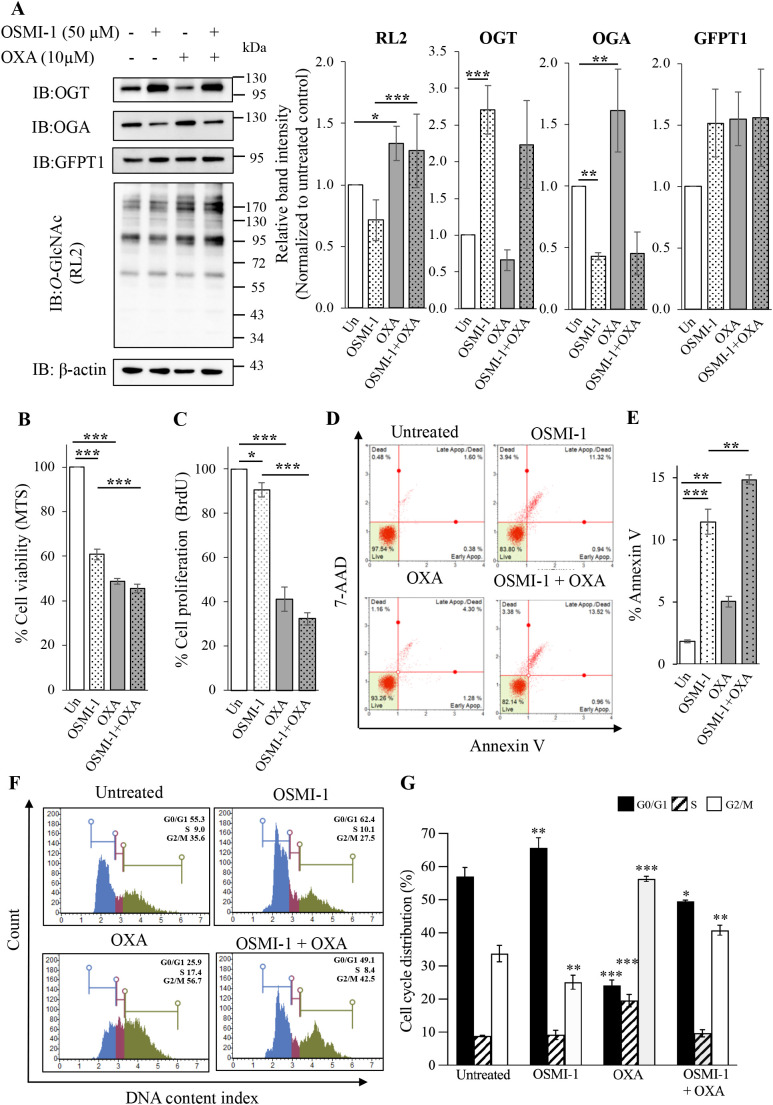
*O*-GlcNAcylation and biological effects of SW620 cells treated with OXA and OSMI-1. (A) Representative IB of *O*-GlcNAcylation (RL2), OGA, OGT, GFPT1, and β-actin and its relative levels (3 independent replicates). (B and C) Bar graph represents the cell viability and cell proliferation of SW620 cells treated with OXA and OSMI-1, respectively (3 independent replicates). (D and E) Representative apoptosis profiles and bar graph represent the total apoptosis (7-ADD + /- and Annexin V+) (3 independent replicates). (F and G) Representative cell cycle profiles and histograms (3 independent replicates). Cells were pretreated with 10 µM OSMI-1 for 1 hour and followed by 48 hours of 10 µM OXA treatment. Cell viability, proliferation, apoptosis, and cell cycle distribution were performed as described in the method. Data are presented as the relative percentage normalized by the untreated control (Un), and values are expressed as the mean ± standard deviation from at least three independent experiments as indicated. *P < 0.05, **P < 0.01, and ***P < 0.001.

Next, we asked if a decreased *O*-GlcNAcylation may lower cell viability of SW620 treated with OXA. OSMI-1, an OGT inhibitor, was used in this study. Our preliminary data showed that treatment of 50 µM OSMI-1 showed a modest reduction of *O*-GlcNAcylation level in comparison to that of lower doses (S2 Fig in S1 Data). Thus, SW620 cells were pretreated with 50 µM OSMI-1 for 1 hour, followed by treatment of 10 µM OXA for 48 hours. OXA treatment significantly increased *O*-GlcNAcylation and OGA but decreased in OGT levels, while OSMI-1 treatment showed a slight decrease in *O*-GlcNAcylation but substantially and significantly decreased in OGA and increased in OGT levels in comparison to those of the untreated control (Fig 2A). In addition, we found that the level of GFPT1, the rate-limiting enzyme that converts fructose-6-phosphate to glucosamine-6-phosphate and enters in the HBP, was increased (Fig 2A). This may lead to an increase of *O*-GlcNAcylation level. Combined treatment of OSMI-1 and OXA showed lesser degree of OGA and OGT expression changes when compared to such a single treatment (Fig 2A). The combined treatment of OSMI-1 and OXA also significantly exhibited a greater decrease in cell viability and proliferation compared to those of OSMI-1-treated or OXA-treated groups (Fig 2B and 2C). Moreover, treatment of OXA and OSMI-1 induced apoptosis and the combined treatment showed a significantly greater increase in apoptosis compared to those of a single treatment (Fig 2D and 2E). Because OXA treatment resulted in only 5% apoptosis, while it led to about 50% reduction of cell viability and proliferation, OXA may also inhibit cell growth. To end this, cell cycle distribution was performed. We found that OXA significantly caused cell cycle arrest at S and G2/M phrases, while OSMI-1 affected at GO/G1phrase, whereas the combined treatments majorly influenced at G2/M phrase (Fig 2F and 2G). In addition, we performed the viability test measured by trypan blue exclusion assay and found that OXA treatment strongly inhibited cell growth, while moderately induced cell death (S3 Fig in S1 Data). These data indicate that the major effect of OXA caused cell growth inhibition through the cell cycle arrest rather than cell death.

### 3.3. Effects of OXA treatment and OGT knockdown

**Fig 3:**
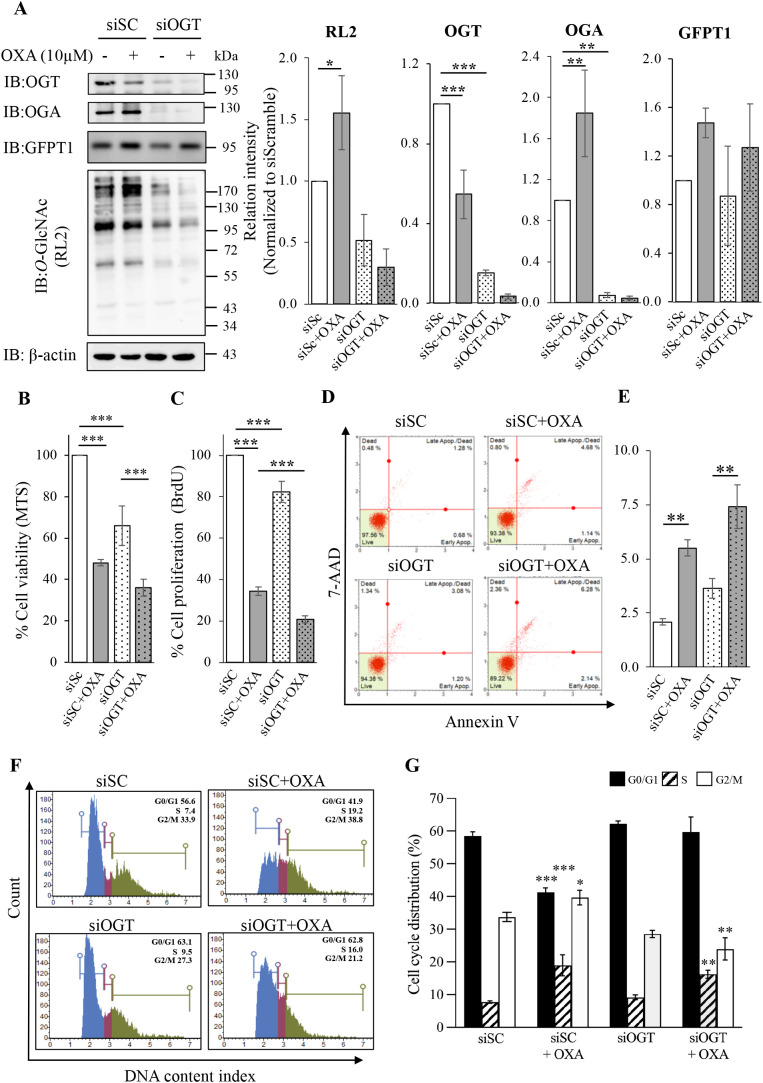
*O*-GlcNAcylation and biological effects of SW620 cells treated with OXA and OGT knockdown. (A) Representative IB of *O*-GlcNAcylation (RL2), OGA, OGT, GFPT1, and β-actin and its relative levels (3 independent replicates). (B and C) Bar graph represents the cell viability (4 independent replicates) and cell proliferation (3 independent replicates) of SW620 cells treated with OXA and OGT knockdown, respectively. (D and E) Representative apoptosis profiles and bar graph represent the total apoptosis (7-ADD + /- and Annexin V+) (3 independent replicates). (F and G) Representative cell cycle profiles and histograms (3 independent replicates). Cells were pretreated with siOGT or siScramble (siSC) for 48hour and followed by 48 hours of 10 µM OXA treatment. Cell viability, proliferation, apoptosis, and cell cycle distribution were performed as described in the method. Data are presented as the relative percentage normalized by the untreated control, and values are expressed as the mean ± standard deviation. *P < 0.05, **P < 0.01, and ***P < 0.001.

Consequently, we asked if a decreased OGT level may lower cell viability of SW620 treated with OXA. Thus, OGT knockdown (siOGT) by RNA interference was performed in this study. After 48 hours of knockdown, siOGT and siScramble cells were treated with 10 µM OXA for 48 hours. Again, OXA treatment increased the levels of *O*-GlcNAcylation and GFPT1 in the siScramble-cells (siSC). Similar to the previous result, OXA treatment also led to an increase in OGA but a decrease in OGT levels. As expected, OGT and *O*-GlcNAcylation levels were lower in the siOGT cells compared to those in the siSC cells (Fig 3A). Interestingly, siOGT cells caused a reduction of OGA level in both with and without OXA treatments. This is likely to be a compensation response when the OGT level was reduced. The combined treatment of siOGT and OXA revealed a significantly greater decrease in cell viability and proliferation compared to those of siOGT or OXA-treated groups (Fig 3B and 3C). In addition, the combined treatment of siOGT and OXA significantly increase in apoptosis compared to that of the siOGT treated group and tended to increase when compared to the siSC + OXA group (Fig 3D and 3E). Similar to the previous experiment of OSMI-1, OXA significantly caused cell cycle arrest at S and G2/M phrases and siOGT-treated cells tended to affect at G0/G1 phrase, whereas the combined treatments majorly influenced at S and G2/M phrase (Fig 3F and 3G). In addition, treatment of OXA at 1 µM OXA for 48 hours was also performed. We found that the combined treatment of siOGT and OXA at lower dose still showed a decrease in cell viability and proliferation although it was lesser effect when compared to those of 10 µM OXA (S4 Fig in S1 Data and Fig 3B and 3G). Furthermore, we performed a clonogenicity of SW620 cells treated with OXA, OGT knockdown, and found that OXA, and OGT knockdown inhibited colony formation and the combination treatment showed a greater effect at both 1 and 10 µM OXA (S5 Fig in S1 Data).

### 3.4. Quantitative proteomics and bioinformatics analysis of SW620 cells treated with OXA and OGT knockdown

**Fig 4:**
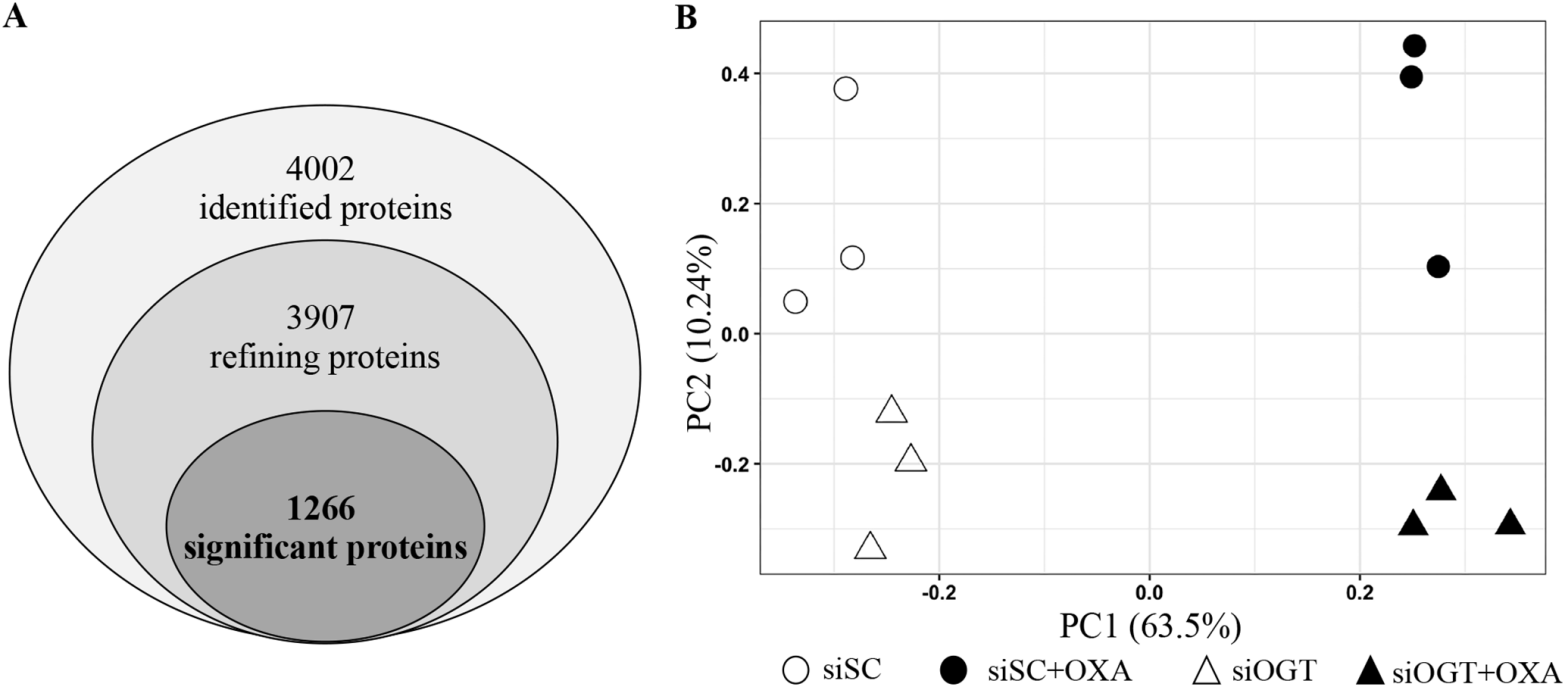
Protein identification and principal component analysis (PCA) of SW620 cells treated with OXA and OGT knockdown. (A) Infographic of proteins identified by Mass spectrometry based-proteomics. (B) PCA score plot of 1,266 proteins discriminated between OXA treatment and OGT knockdown. Proteins obtained from SW620 cells treated with (1) siSC (2) siSC + OXA, (3) siOGT, and (4) siOGT + OXA in three biological replicates (a total of 12 samples) were analyzed by mass spectrometer.

Since OGT knockdown had a greater effect to reduce *O*-GlcNAcylation, we chose this technique to find potential target proteins affected by reducing *O*-GlcNAcylation and OXA treatment in SW620 cells. To end this, mass-spectrometry based proteomics and a label-free quantitative analysis were performed. Four groups including (1) siScramble (siSC), (2) siSC + OXA, (3) siOGT, and (4) siOGT + OXA in three biological replicates, resulting in a total of 12 LC-MS/MS runs, were used in this study. A total of 3,907 proteins (refining) among all samples were identified and their relative protein expressions were analyzed (Fig 4A and S3 Data). Among them, statistical analysis (One-way ANOVA) showed that 1,266 proteins were significantly different at least one among all groups. Of 1,266 proteins, principal component analysis (PCA) reveals that principal component 1 (PC1) clearly discriminates between the OXA-treated and the absence of OXA groups, while principal component 2 (PC2) apparently segregates between the siOGT-treated and the siSC-treated groups (Fig 4B and the Supplementary data S3_Proteomics in S3 Data).

**Fig 5:**
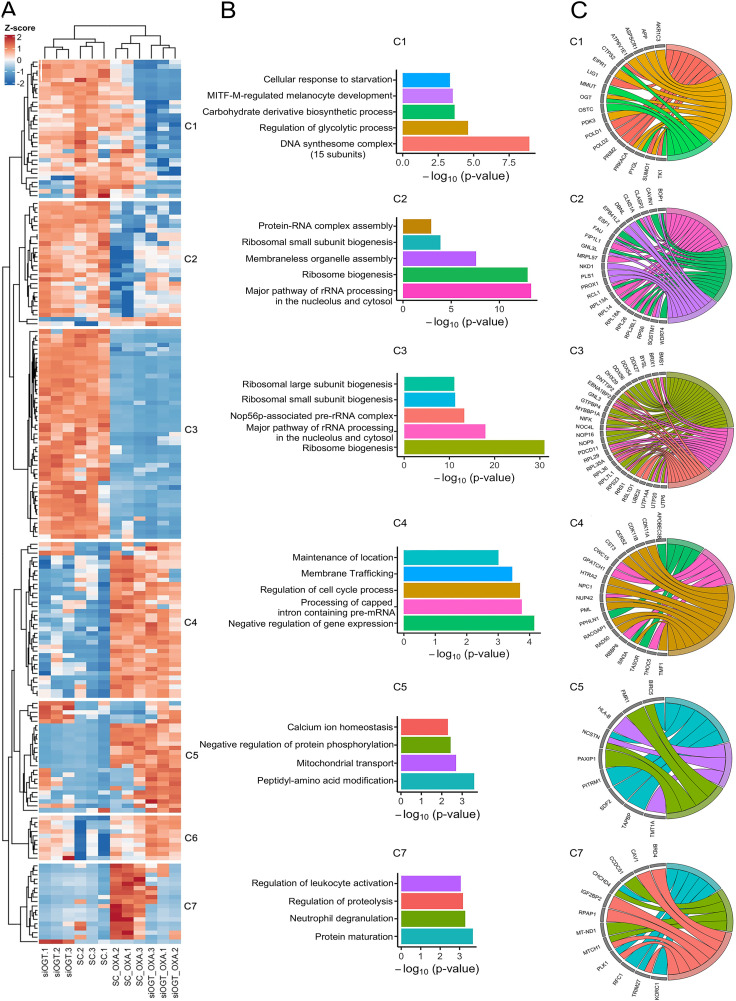
Protein heatmap and clustering of the top 100 proteins by PC1 and PC2 analysis of SW620 cells affected by OXA and OGT knockdown. Proteins obtained from SW620 cells treated with (1) siSC (2) siSC + OXA, (3) siOGT, and (4) siOGT + OXA in three biological replicates were analyzed by mass spectrometer. (A) A heatmap of differentially expressed proteins grouped into 6 clusters analyzed by R. (B) Representative graphs of enriched ontology analysis in 6 clusters analyzed by Metascape. (C) GO chord diagrams reveal proteins in the most top 3 enriched terms in 6 clusters..

We further investigated the biological significance of the identified proteins by selecting the top 100 proteins that contributed most to PC1 and PC2 (union) and subjected this combined set to bioinformatics analysis. A heatmap of 194 proteins with hierarchical clustering clearly revealed the effects of OXA and OGT knockdown (Fig 5A). Proteins in each cluster were analyzed by bioinformatics analysis using Metascape database and the most top 3 enriched terms in 6 clusters were visualized by GOChord (Fig 5B and 5C, and Supplementary data S4_Bioinformatics S4 Data).

Hierarchical clustering of proteins with differential expression following OXA treatment revealed several functional groups based on their regulation across the siOGT and siSC conditions and was majorly clustered into two main groups: downregulated proteins by OXA (Clusters 1, 2, and 3) and upregulated proteins by OXA (clusters 4, 5, and 7).

**Fig 6:**
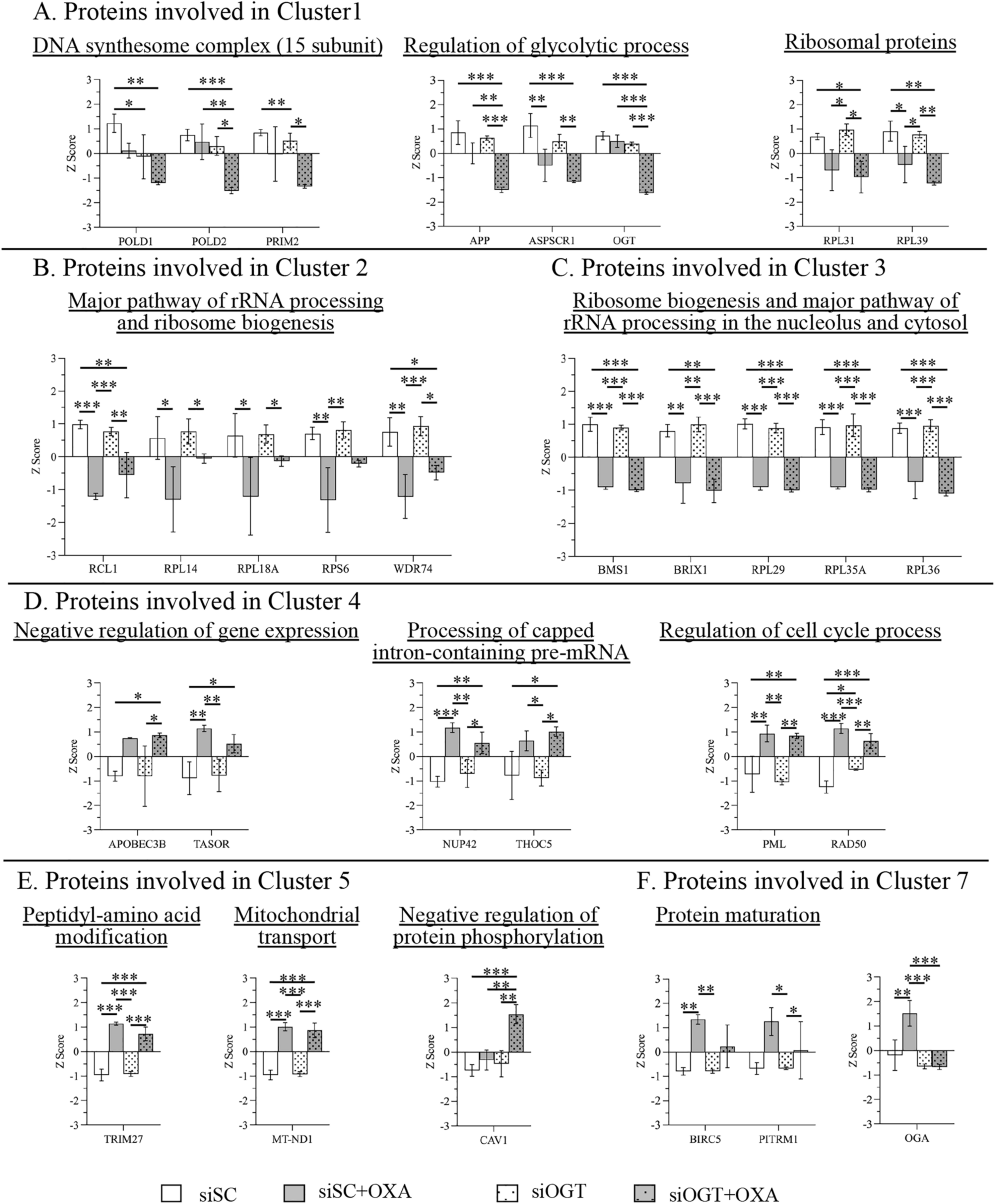
The levels of differentially expressed proteins by treatment of OXA and OGT knockdown. (A-C) Biological functions and downregulated proteins by OXA and siOGT treatment in cluster 1, 2, and 3; (D-F) Biological functions and upregulated proteins by OXA and siOGT treatment in cluster 4, 5, and 7. Protein expression levels were obtained from the label-free quantitative mass-spectrometry data of three independent experiments of four samples including (1) siSC (2) siSC + OXA, (3) siOGT, and (4) siOGT + OXA. Data are presented as the mean z score ± standard deviation. *P < 0.05, **P < 0.01, ***P < 0.001.

Cluster 1 was dominant in the siOGT group which were involved in (1) DNA synthesome complex (15 subunits) (CORUM:1108) such as POLD1, POLD2, PRIM2; and (2) regulation of glycolytic process (GO:0006110) such as APP, ASPSCR1, and OGT (Fig 6A). Cluster 2 was dominant in the siSC group which were involved in (1) major pathway of rRNA processing (R-HAS-6791226) and (2) ribosome biogenesis (GO:0042254). Some of them were RCL1, RPL14, RPL18A, RPS6, and WDR74 (Fig 6B). Cluster 3 demonstrated the downregulated proteins dominantly in both siSC + OXA and siOGT + OXA groups and they were strongly involved in ribosome biogenesis (GO:0042254) and major pathway of rRNA processing in the nucleolus and cytosol (R-HSA-6791226). Some of them were BMS1, BRIX1, RPL29, RPL35A, and RPL36 (Fig 6C).

Conversely, cluster 4 revealed the upregulated proteins dominantly in both siSC + OXA and siOGT + OXA groups which were involved in (1) negative regulation of gene expression (GO:0045814) such as APOBEC3B and TASOR; (2) processing of Capped Intron-Containing Pre-mRNA (R-HSA-72203) such as NUP42 and THOC5; and (3) regulation of cell cycle process (GO:0010564) such as PML and RAD50 (Fig 6D). Cluster 5 showed the upregulated proteins dominantly in the siOGT + OXA group which were involved in peptidyl-amino acid modification (GO:0018193) such as TRIM27, mitochondrial transport (GO:0006839) such as MT-ND1, and negative regulation of protein phosphorylation (GO:0001933) such as CAV1 that was dominantly in the siOGT group (Fig 6E). Cluster 7 revealed the upregulated proteins dominantly in the siSC + OXA group which were involved in protein maturation (GO:0051604), and regulation of proteolysis (GO:0030162) such as BIRC5 and PITRM1 (Fig 6F). Cluster 6 did not show any significant protein-protein interaction enrichment.

To validate proteins obtained by the proteomics data and identify potential pathways of the combined treatment of OXA and OGT knockdown, we selected ribosomal protein S6 (RPS6), a key protein component of the ribosome and a downstream target of mTOR signaling pathway. Our proteomics data revealed that the levels of RPS6 and mTOR were down in both siSC + OXA and siOGT + OXA (Fig 6B and the Supplementary data S3_Proteomics “quantify sheet in S3 Data). To end this, we performed immunoblotting. The results showed that OXA treatment decreased the levels of mTOR as well as RPS6 and the phosphorylation of RPS6, especially in the combination with OGT knockdown (S6 Fig in S1 Data). However, there was no change or a slight effect of the proteins in mTOR pathway in OGT knockdown alone without OXA treatment (S6 Fig in S1 Data).

## 4. Discussion

*O*-GlcNAcylation is a dynamic post-translational modification (PTM) of proteins that plays a vital role in cellular signaling, and its aberrant modification is implicated in various diseases including cancers [21,22]. When cancer cells face cellular stress, such as exposure to chemotherapy drugs, global *O*-GlcNAcylation levels increase dramatically, a mechanism believed to facilitate survival and cellular reprogramming [23]. This heightened *O*-GlcNAcylation enhances chemoresistance, suggesting that interventions targeting this modification could offer a strategy to overcome treatment failure [13,24]. Despite this evidence, the specific molecular mechanisms governing the interplay between chemotherapy response and *O*-GlcNAcylation in cancer cells have yet to be fully elucidated.

In the present study, we demonstrate that treatment with OXA, a first-line chemotherapy drug for patients with metastatic CRC, increased the *O*-GlcNAcylation level in SW620 metastatic CRC cells. This finding is consistent with previous research showing that various chemotherapeutic drugs, including Doxorubicin (DOX) and Camptothecin (CPT), elevate *O*-GlcNAcylation level across multiple cancer cell lines (e.g., MCF-7, HL60, HeLa) [24]. Importantly, prior studies also report that blocking of *O*-GlcNAcylation activity or reduction of OGT level enhances the chemotherapeutic responses [24]. Furthermore, studies utilizing *in vivo* mouse models confirm that the *O*-GlcNAcylation pathway is critical for promoting tumor survival and chemotherapy resistance. Genetic knockout of the rate-limiting HBP enzyme, GFPT1 inhibited *O*-GlcNAcylation, induces ferroptosis, and mitigates chemoresistance of orthotopic bladder cancer in Gfpt1^-/-^ mice [25]. The *O*-GlcNAc modification of NRF2 was found to promote malignancy and cisplatin resistance in both *in vitro* and xenograft mouse models [26]. The upregulation of HBP caused stabilization of YAP via *O*-GlcNAcylation and this modification subsequently promotes tumor growth and chemotherapy resistance in mouse models [27]. These results collectively indicate that chemotherapeutic drugs promote a survival signal via *O*-GlcNAcylation, suggesting that reducing *O*-GlcNAc level may enhance the beneficial therapeutic effects of these drugs.

Our results demonstrate that decreasing *O*-GlcNAcylation via the OGT inhibitor OSMI-1 or OGT knockdown significantly increases the sensitivity of SW620 metastatic CRC cells to OXA treatment, resulting in a higher rate of apoptosis compared to either single treatment. These findings are consistent with established literature showing that blocking OGT activity synergistically enhances the effect of other chemotherapeutic agents across various cancers. OSMI-1 enhanced the anticancer effects of regorafenib in CRC cells [28] and synergistically promoted doxorubicin-induced apoptosis in HepG2 cells by modulating Bcl-2 and p53 pathways [29]. In addition, increasing *O*-GlcNAcylation enhanced OXA resistance in SW480 cells, while reducing the modification decreased OXA resistance in HCT116 cells [30]. Furthermore, reducing OGT expression increased the sensitivity of bladder cancer cells to cisplatin [31], gemcitabine, and paclitaxel [32]. Collectively, this evidence strongly suggests that blocking OGT activity or reducing OGT expression is a viable strategy to enhance cell death, induce apoptosis, and overcome chemoresistance in multiple cancer types.

In the present study, we also found that interference with *O*-GlcNAcylation of SW620 cells by the OGT inhibitor or OGT knockdown has profound effects on *O*-GlcNAc cycling enzymes in terms of OGT and OGA expression. OSMI-1 treatment increased in OGT but decreased in OGA protein expression. OGT knockdown lead to a dramatic reduction of both OGT and OGA protein expression levels (Fig 3A and 6A and 6F). Multiple studies confirm an interplay of cycling enzymes, OGT and OGA, controlling the *O*-GlcNAcylation, which is essential for maintaining cellular homeostasis. Chemical inhibition of OGA using TMG increased the OGA protein level in SH-SY5Y neuroblastoma [33], while OGT using OSMI-1 induced OGT protein level in prostate cancer cells [34]. Conversely, genetic depletion of OGT consistently resulted in the downregulation of OGA abundance [35,36]. Consistent with these data, our results imply that cells perform compensatory adaptation by modulating OGT/OGA protein expression to restore optimal *O*-GlcNAcylation levels. We note that the distinction between OGT activity blockade (inhibitor) and OGT gene suppression (knockdown) necessitates further investigation to fully define the resultant cellular effects.

Mass spectrometry-based proteomics and bioinformatics analysis identified several proteins and pathways altered by the combined effect of OXA and OGT knockdown in SW620 cells. Firstly, OXA induces ribosomal stress. A major finding was the downregulation of numerous ribosomal proteins (Cluster 1, 2, and 3) following OXA treatment. This aligns with external studies demonstrating that OXA primarily induces ribosome biogenesis stress by inhibiting ribosomal RNA synthesis, rather than solely relying on DNA damage [37,38]. Secondly, OGT is associated to ribosomal protein expression. While OGT knockdown alone did not affect ribosomal protein expression under normal conditions, its combination with OXA revealed a complex dependence. The expression of certain ribosomal proteins (e.g., RPL39, RPL14) was partially dependent on OGT and *O*-GlcNAcylation, as OGT reduction amplified the OXA-induced downregulation (e.g., RPL39 showed a dominant effect in the siOGT + OXA group). Other ribosomal proteins (e.g., RPL29, RPL35A) were downregulated by OXA irrespective of OGT status. These findings combined with prior research showing that ribosomal proteins are targets for *O*-GlcNAcylation, which regulates ribosome biogenesis and translation [36,39] indicate that ribosomal proteins are major functional targets of both OXA and the *O*-GlcNAcylation pathway. This suggests that *O*-GlcNAcylation may contribute to ribosomal synthesis and stability in the face of stress. Further research into the *O*-GlcNAc modification of ribosome-associated proteins during proteotoxic stress is warranted.

Ribosomal proteins (RPs) are essential for stabilizing rRNA structures and facilitating rRNA processing, playing critical roles in ribosome biogenesis and function [40]. Among these, RPS6 is phosphorylated via the activation of the mTOR signaling pathway to promote the synthesis of the 40S ribosomal subunit. The mTOR pathway serves as a primary regulator of pre-rRNA transcription and rRNA synthesis, specifically through S6K1-mediated phosphorylation of RPS6 (pRPS6) [41]. Our study demonstrated that OXA decreased the expression of both mTOR and RPS6, as well as overall RPS6 phosphorylation. While the reduction in mTOR and RPS6 levels appears substantial, the specific contribution of OXA alone versus the combination of OXA and OGT knockdown in decreasing RPS6 phosphorylation remains to be fully elucidated. Therefore, further studies are required to precisely determine whether OGT reduction interacts with RPS6 and its phosphorylation via mTOR signaling.

In addition, the combined treatment of OXA and OGT knockdown dominantly downregulated proteins within the DNA synthesome complex (Cluster 1, POLD1, POLD2, and PRIM2), which are crucial for DNA replication. These proteins, components of the DNA polymerase and primase complexes, were significantly reduced. This is notable as PRIM2 and POLD1 are frequently upregulated and aberrantly expressed in CRC and other cancers [42,43]. Furthermore, POLD1 has previously been shown to be associated with OGT in the cellular response to oxidative stress [44]. Moreover, amyloid-beta precursor protein (APP) was downregulated in OGT knockdown and OXA treatment. The increased expression of APP was shown in multiple types of cancer and its expression may control cancer cell proliferation [45]. APP was reported to be modified by *O*-GlcNAc at threonine 576 and its modification may regulate trafficking and processing [46]. The strong downregulation of these DNA replication and proliferation-associated proteins highlights a likely mechanism by which OGT reduction enhances the efficacy of OXA. Thus, further studies are needed to identify precisely *O*-GlcNAc modified proteins and OGT-dependent signaling pathways which may directly or indirectly regulate DNA replication upon the chemotherapeutic responses.

In contrast, OXA treatment led to the upregulation of proteins involved in gene expression (e.g., APOBEC3B, TASOR) and cell cycle regulation (e.g., PML, RAD50). Apolipoprotein B mRNA editing enzyme catalytic subunit 3B (APOBEC3B) is reported to promote tumor development [47], and DNA repair protein RAD50 (RAD50) is reported to promote ovarian cancer progression [48]. Significantly, OGT knockdown combined with OXA treatment dominantly upregulated Caveolin-1 (CAV1). CAV1, a key structural protein of caveolae, is known to be modified by *O*-GlcNAc, and this modification is implicated in controlling tumor growth [49]. These findings highlight that OGT knockdown alters the expression of specific proteins involved in gene regulation and proliferation during OXA treatment. However, further mechanistic studies are necessary to clarify precisely how OGT modulates these proteins to affect CRC chemotherapeutic response.

Lastly, OGT knockdown likely perturbs cellular signaling beyond *O*-GlcNAcylation due to the extensive crosstalk this modification shares with other PTMs, particularly phosphorylation and *N*-glycosylation. *O*-GlcNAcylation and phosphorylation engage in dynamic regulation, often competing for shared sites or exerting synergistic control over proximal or distant sites on target proteins to regulate signaling, metabolism, and gene expression [7,50]. Furthermore, OGT knockdown has been specifically linked to a decrease in high mannose N-glycans via increased FOXO3 phosphorylation and reduced MAN1A1 expression, which ultimately suppressed metastasis in cholangiocarcinoma cells [51]. Consequently, a comprehensive investigation into the effects of altering *O*-GlcNAcylation is necessary to fully map its broad regulatory impact across the cellular landscape.

This study has two primary limitations: first, the use of a single cell line (SW620) restricts the generalizability of the findings to other CRC cell types; second, the absence of *in vivo* data limits the determination of effective drug doses and the translational relevance to clinical treatment. Future studies using diverse CRC cell lines and animal models are essential to substantiate the efficacy of this combination drug therapy.

In conclusion, this study demonstrates the critical role of OGT and protein *O*-GlcNAcylation in the response of SW620 metastatic CRC cells to OXA treatment. We found that combining OXA with either OGT inhibition or knockdown significantly enhances chemotherapeutic sensitivity by suppressing cancer cell growth, inducing apoptosis, and causing cell cycle arrest. Proteomic analysis identified a key group of altered proteins, predominantly involved in ribosomal biosynthesis and metabolism, which are affected by OXA and OGT interference. Furthermore, the pathways identified through this combination treatment provide crucial insights into the mechanisms underlying OXA resistance and offer potential therapeutic targets to improve OXA-based regimens. Future work should focus on validating these findings in other CRC cell lines and diverse cancer types to fully establish the clinical utility of targeting *O*-GlcNAcylation to enhance chemotherapy efficacy.

## Supporting information

S1 Data (S1–S6 Figs). (PDF)

S2 Raw Images Raw images underlying all immunoblot results (S7–S18 Figs). (PDF)

S3 Data Lists of proteins identified and quantified by label-free quantitative mass spectrometry analysis. (XLSX)

S4 Data Lists of proteins identified by bioinformatics analysis using Online-Metascape database. The raw MS and label-free quantitative proteomics data have been deposited to the JPOSTrepo (Japan ProteOme Standard Repository) with the dataset identifier (https://repository.jpostdb.org/preview/1846806717684648876063a and Access key: 4902).
(XLSX)
