# Supplementary material for: Reducing OGT and O-GlcNAcylation enhance the anticancer effects of oxaliplatin in SW620 metastatic colorectal cancer cells
Source: PLoS One. 2026 Feb 10;21(2):e0341971. doi: 10.1371/journal.pone.0341971 (PMC12890163; doi:10.1371/journal.pone.0341971)
Supplement: S1 Data — (PDF) [file pone.0341971.s001.pdf]

Supplementary Data

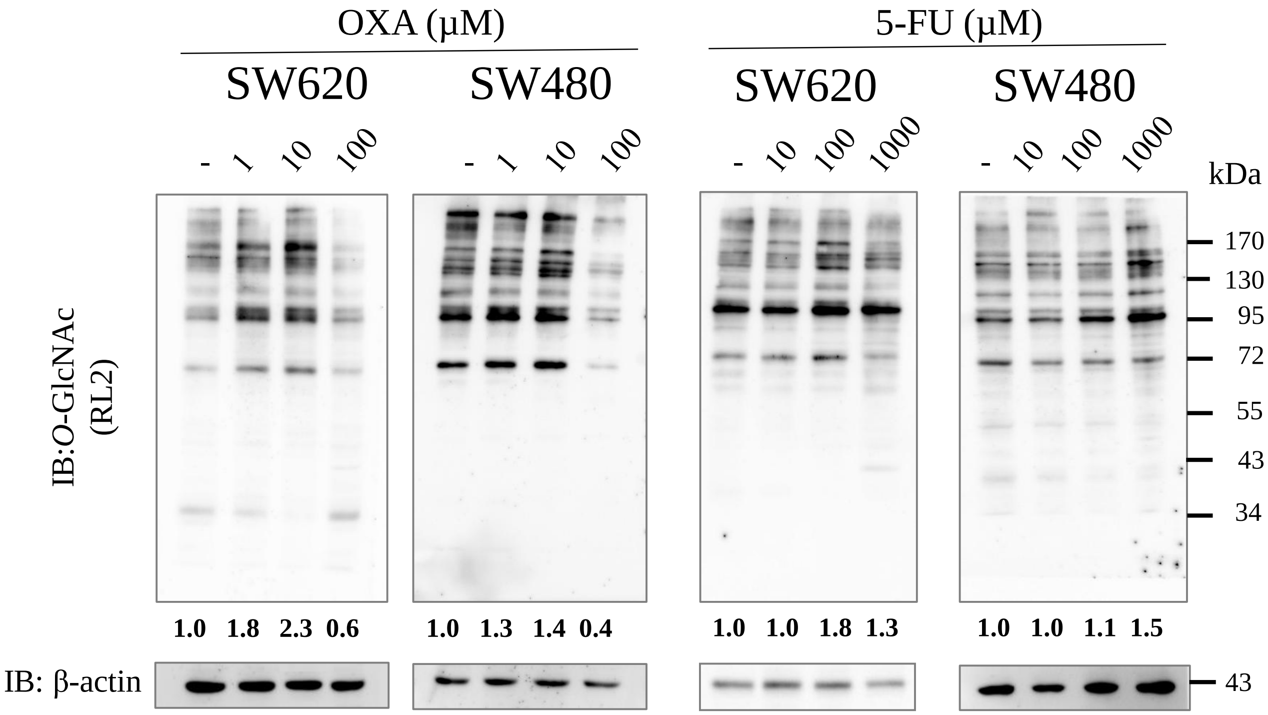

Figure S1. *O*-GlcNAcylation level of SW480 and SW620 cells treated with OXA and 5-FU. Immunoblots (IB) of *O*-GlcNAc modified proteins (RL2) and β-actin. Membranes were firstly probed by RL2 antibody, stripped and re-probed with β-actin antibody as indicated in the figures. Values provided under the immunoblots are the relative protein intensities normalized by those of the untreated cells (-).

Supplementary Data

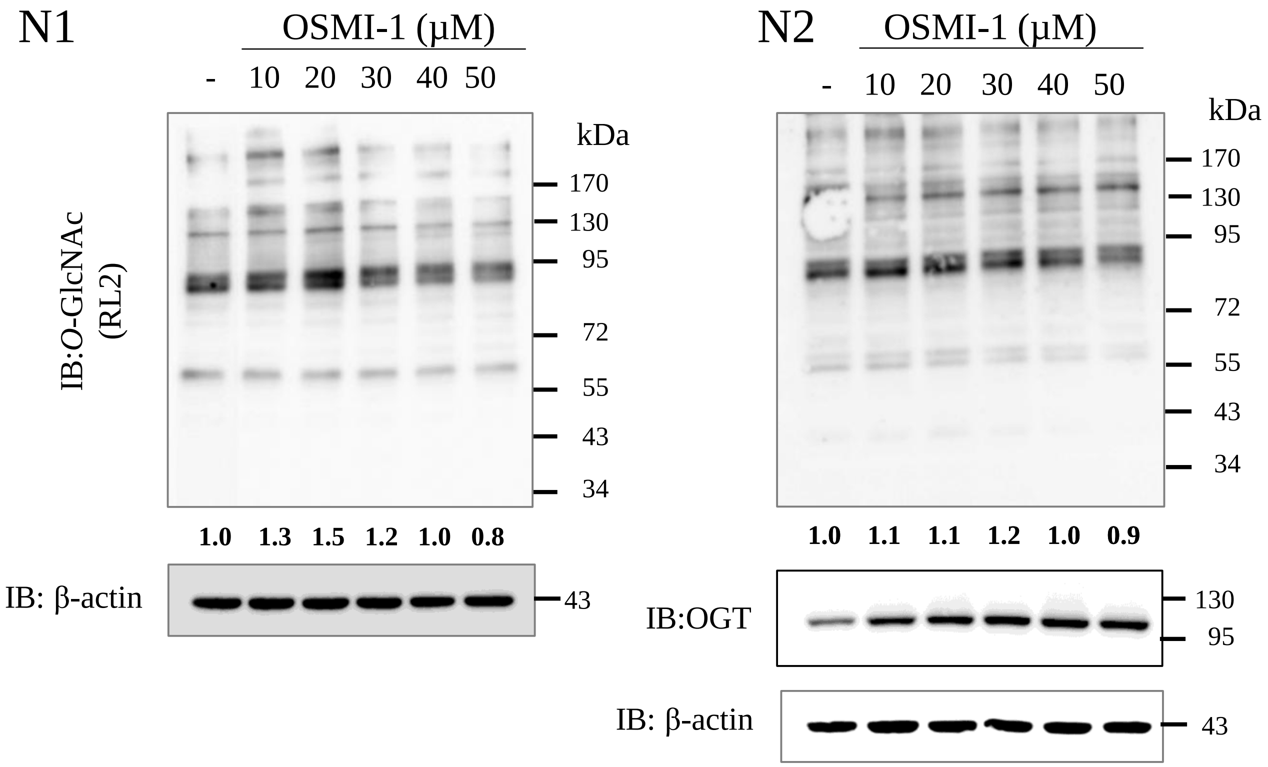

Figure S2. The level of *O*-GlcNAcylation of SW620 cells treated with OSMI-1. Immunoblots (IB) of *O*-GlcNAc modified proteins (RL2) and β-actin of 2 independent replicates. Membranes were firstly probed by RL2 antibody, stripped and re-probed with β-actin antibody as indicated in the figures (OGT was probed with anti-OGT antibody for N2). Values provided under the immunoblots are the relative protein intensities normalized by those of the untreated cells (-).

Supplementary Data

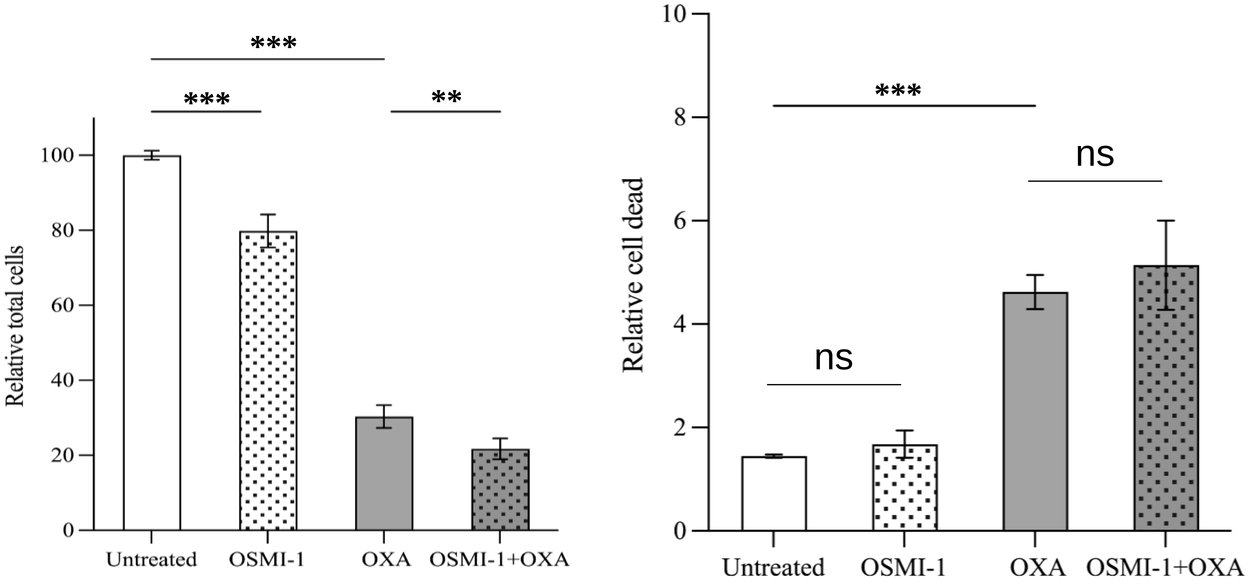

Figure S3. Trypan Blue Assay of SW620 cells treated OSMI-1, OXA, and the combination of OSMI-1 and OXA. SW620 cells were cultured in 12 well plates (100,000 cells/well) for 24 hours. On the next day, cells were treated with OXA (10 µM), OSMI-1 (50 µM), or combination of OXA and OSMI-1 for 48 hours. Then, cells were harvested by trypsinization and cell viability was determined by trypan blue assay. The assay was performed in 2 independent replicates. Values are presented as the mean ± standard deviation $^{**}P<0.01$, $^{***}P<0.001$, and ns = not significant.

Supplementary Data

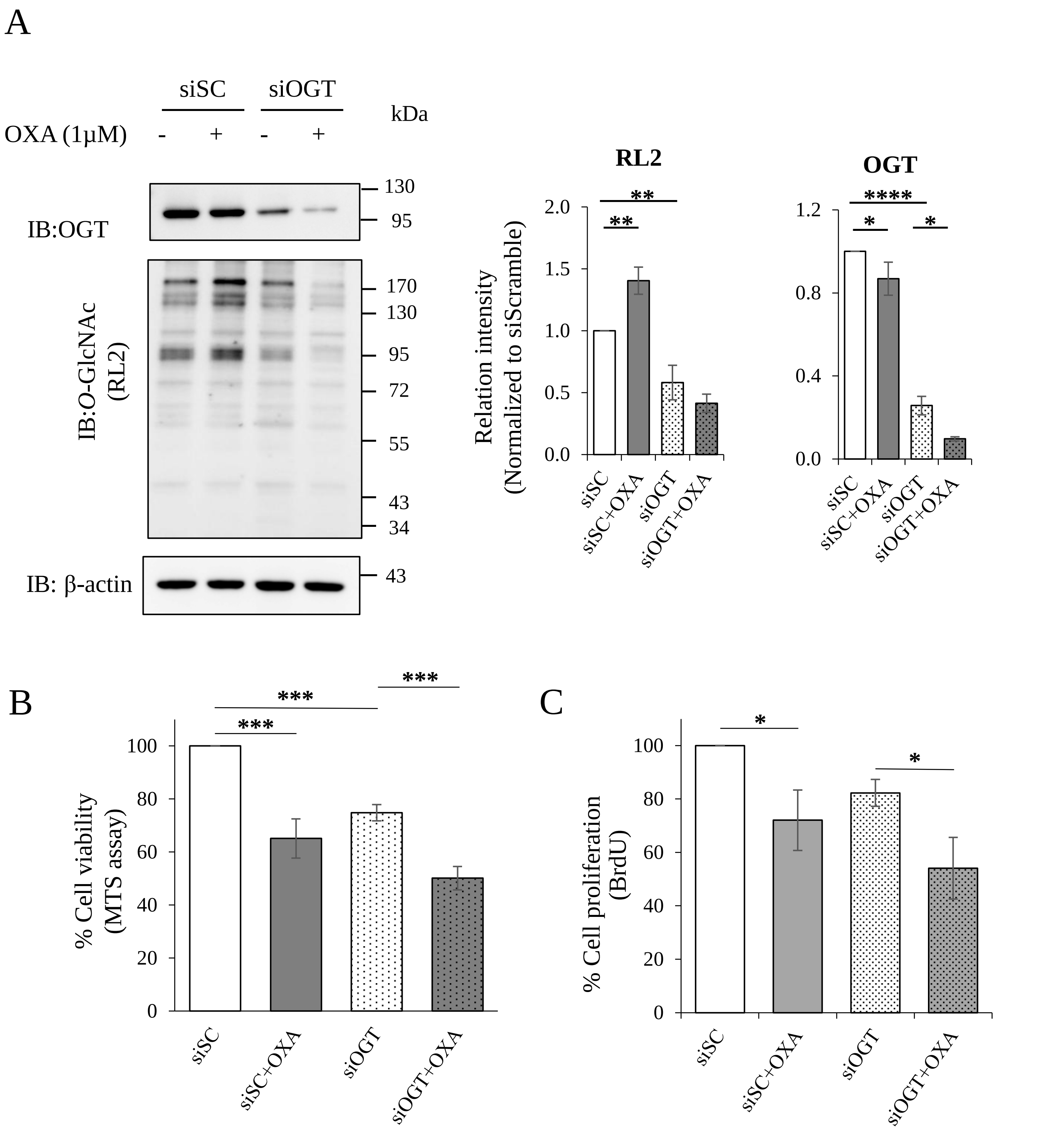

Figure S4. *O*-GlcNAcylation, OGT, and cell viability and proliferation of SW620 cells treated by 1 µM OXA and OGT knockdown. (A) Representative IB of *O*-GlcNAcylation (RL2), OGT, and β-actin and its relative band intensity of RL2 and OGT normalized by β-actin (3 independent replicates). (B) Bar graph represents cell viability of SW620 cells determined by MTS assay (5 independent replicates). (C) Bar graph represents cell proliferation of SW620 cells determined by BrdU assay (3 independent replicates). Cells were treated with siScramble (siSC) or siOGT for 48 hours and followed by 1 µM OXA treatment for 48 hours. Data are presented as the relative percentage normalized by the untreated siScramble control, and values are expressed as the mean ± standard deviation. *p<0.05, **P<0.01, and ***P<0.001.

Supplementary Data

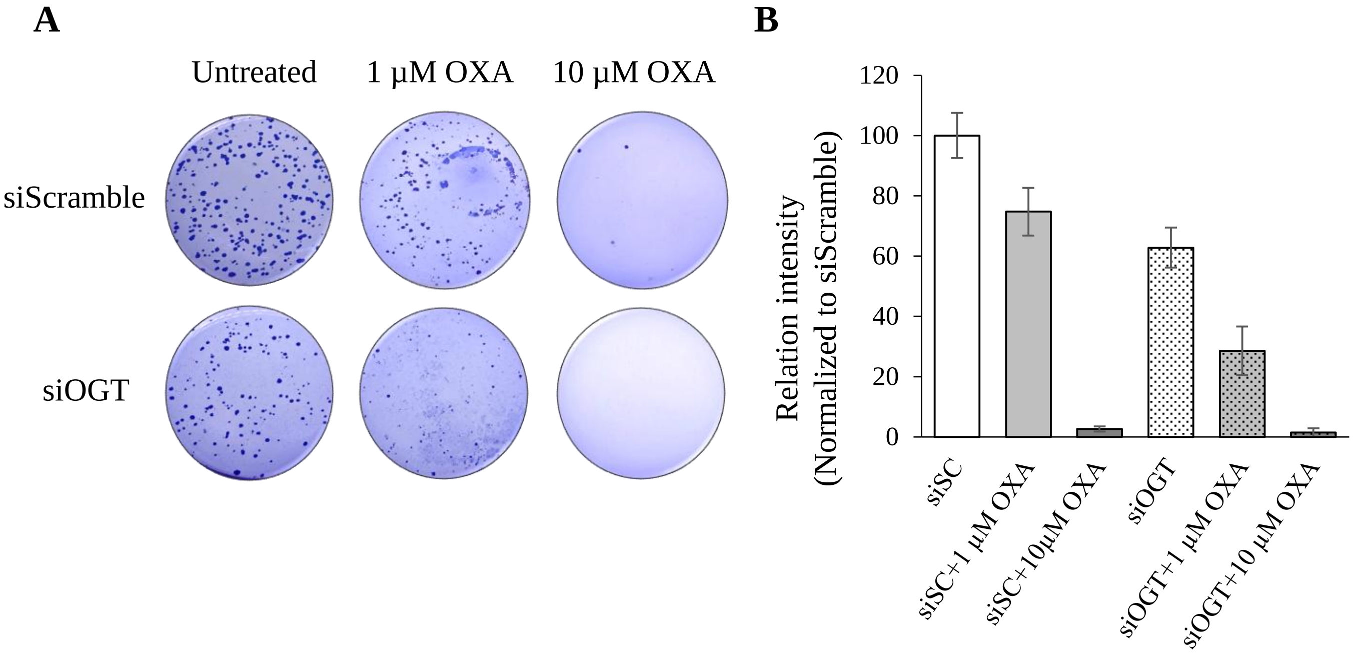

Figure S5. Anchorage-independent cell growth of SW620 cells treated by OXA and OGT knockdown. (A) Representative colony formation in soft agar stained by crystal violet. (B) Graph represents relative colony numbers. SW620 cells were treated with siOGT or siScramble (siSC) for 48 hours and followed by 48 hours of 1 µM or 10 µM OXA treatment. Data are presented as the relative percentage normalized by the untreated siScramble control, and values are expressed as the mean ± standard deviation (2 technical replicates)

Method of Anchorage-independent growth assay. Anchorage-independent cell growth assay *in vitro* was performed using soft agar cultures as described previously with some modifications (42-43). Briefly, $2x10^4$ cells (trypsinized siScramble or siOGT cells knock-downed for 48 hours) were suspended in 1 ml top agar medium (the complete medium with 0.3% agar). The cell suspension was then overlaid onto 1.5 ml bottom agar medium (the complete medium with 0.6% agar) in 6-well culture plates in duplicate. Cells were treated with 0.2% DMSO, 1 µM or 10 µM OXA for 3 days. After 3 days, cells were replaced with the complete medium (no drugs) for every 3 days until 24 days. Cells were then fixed with methanol and stained with 0.0005% crystal violet staining solution. Colony number count was determined by ImageJ software version 2.4.1 (National Institutes of Health, Bethesda, MD, USA).

References

42. Netsirisawan P, Chaiyawat P, Chokchaichamnankit D, Lirdprapamongkol K, Srisomsap C, Svasti J, Champattanachai V. Decreasing *O*-GlcNAcylation affects the malignant transformation of MCF-7 cells via Hsp27 expression and its *O*-GlcNAc modification. Oncol Rep. 2018 Oct;40(4):2193-2205. doi: 10.3892/or.2018.6617. Epub 2018 Aug 1. PMID: 30106436.

43. Liu F, Lv R, Qiao X, Lv G, Yuan H, Han J, Wang X, Wan J, Wang M. Salinomycin and oxaliplatin synergistically enhances cytotoxic effect on human colorectal cancer cells in vitro and in vivo. Sci Rep. 2025 Apr 23;15(1):14056. doi: 10.1038/s41598-025-98633-5. PMID: 40269151; PMCID: PMC12019327.

Supplementary Data

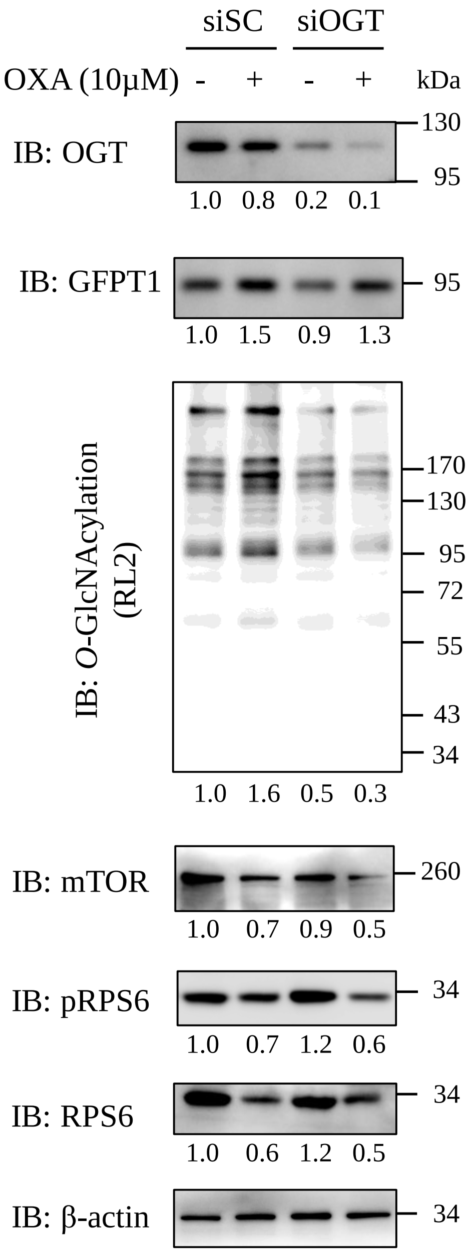

Figure S6. Validation and proposed signaling pathways affected by OXA and OGT knockdown. Representative IB of OGT, GFPT1, *O*-GlcNAcylation (RL2), mTOR, pRPS6, pRPS6 and β-actin. Values provided under the immunoblots are the relative protein levels normalized by those of the siScramble group (-). Cells were pretreated with siOGT or siScramble (siSC) for 48 hour and followed by 48 hours of 10 μM OXA treatment. Data are presented as the relative percentage normalized by the untreated siScramble control, and values are expressed as the mean of at least three independent experiments.
