# Supplementary material for: Reducing OGT and O-GlcNAcylation enhance the anticancer effects of oxaliplatin in SW620 metastatic colorectal cancer cells
Source: PLoS One. 2026 Feb 10;21(2):e0341971. doi: 10.1371/journal.pone.0341971 (PMC12890163; doi:10.1371/journal.pone.0341971)
Supplement: S2 Raw Images — (PDF) [file pone.0341971.s002.pdf]

Supporting information:

All raw images (full size immunoblots) were detected by ImageQuant LAS4000 digital imaging system (GE Healthcare).

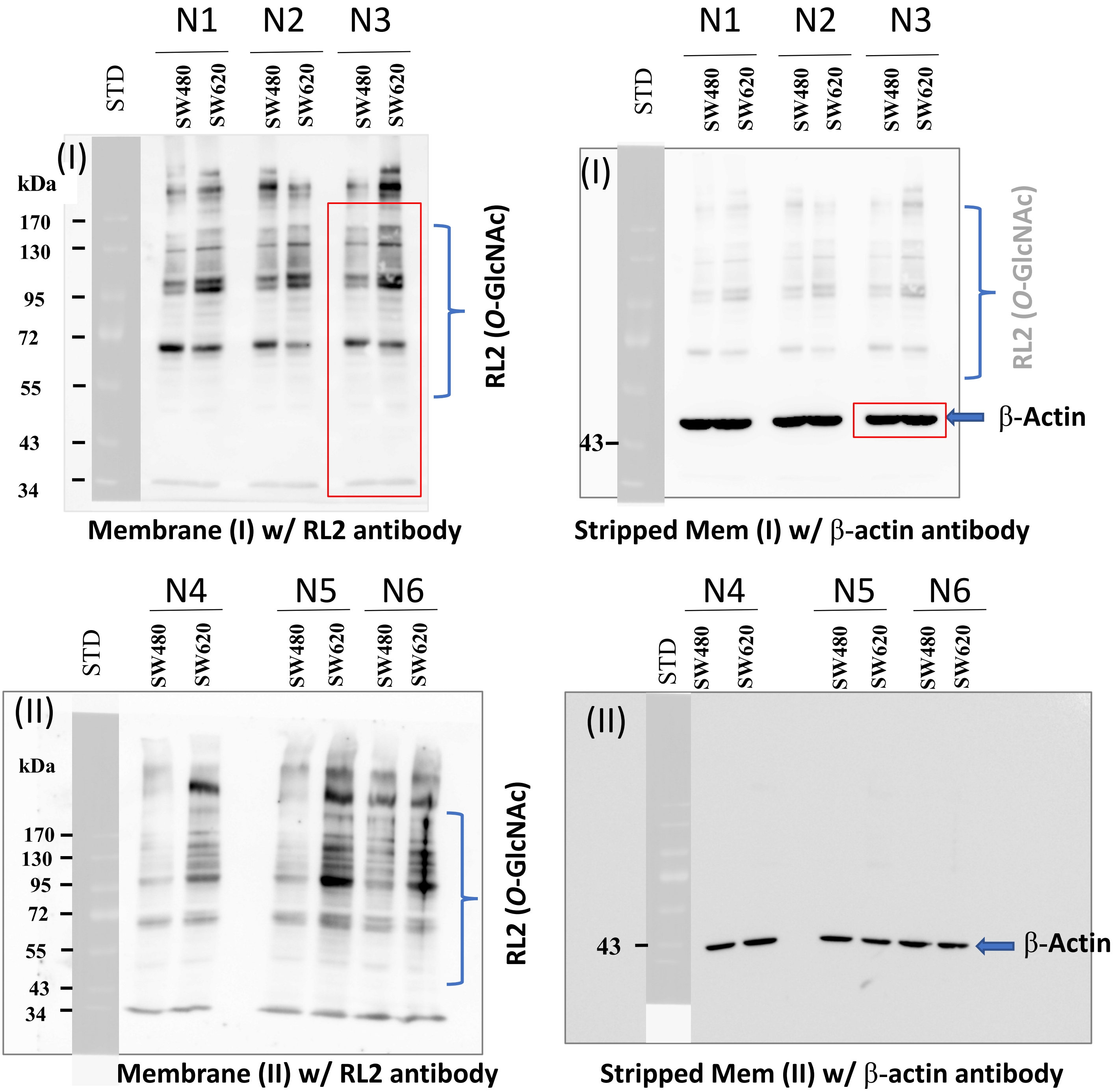

Figure S7. Raw data shown in Figure 1A. *O*-GlcNAcylation level of SW480 and SW620 CRC cells. Immunoblots (IB) of *O*-GlcNAc modified proteins (RL2) and β-actin on Membrane (I and II) of 6 independent experiments, respectively (6 independent replicates). Membranes were firstly probed by RL2 antibody, stripped and re-probed with β-actin antibody as indicated in the figures. Gray texts represent the remaining band intensities of *O*-GlcNAc-modified proteins after stripping. Red boxes are bands showed in the main text.

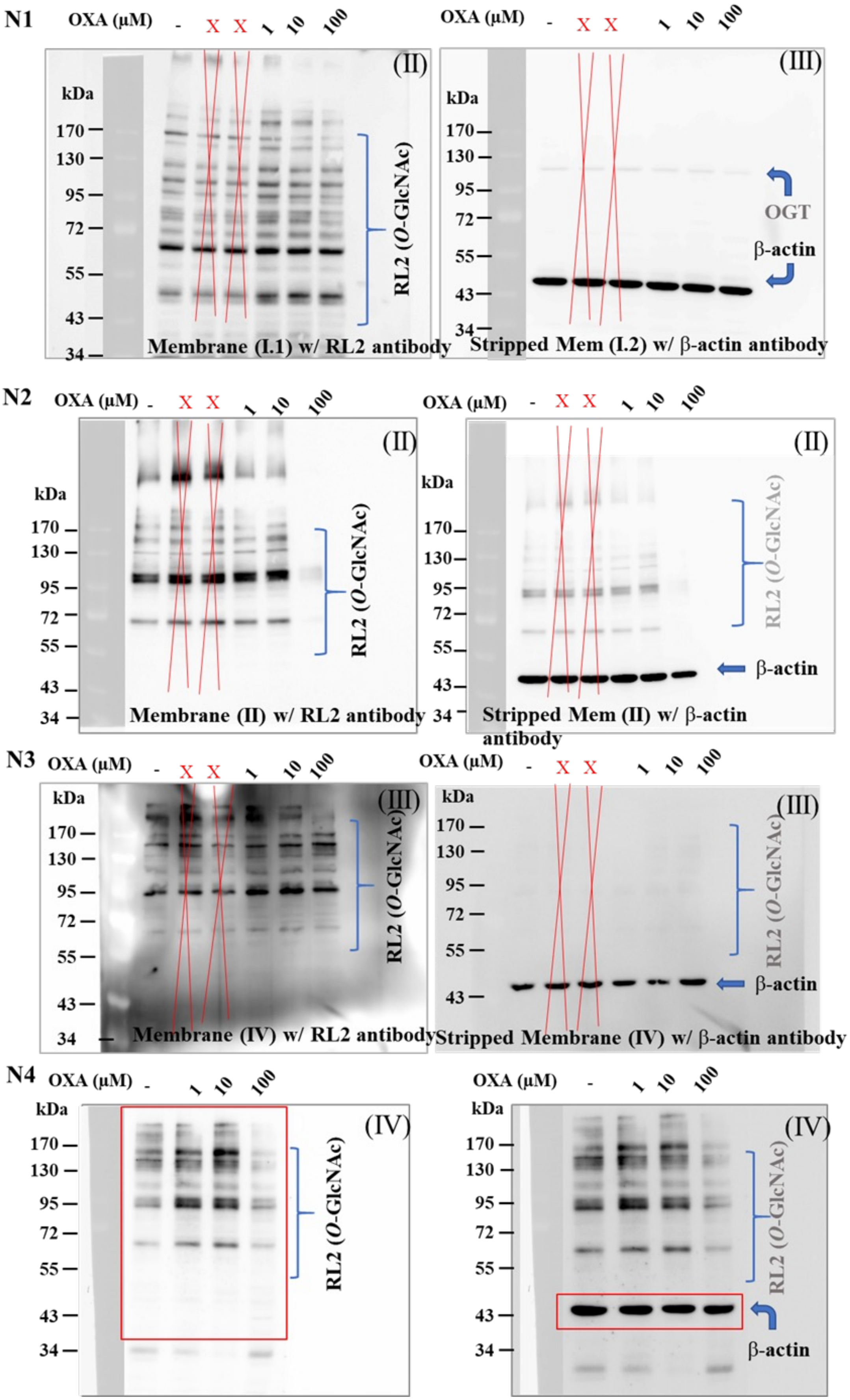

Figure S8. Raw data shown in Figure 1C. *O*-GlcNAcylation level of SW620 cells treated with OXA (1-100 μM, at 48 hours). Immunoblots (IB) of *O*-GlcNAc modified proteins (RL2) and β-actin on Membrane of 4 independent replicates, respectively. Membranes were firstly probed by RL2 antibody, stripped and re-probed with β-actin antibody as indicated in the figures. Gray texts represent the remaining band intensities of *O*-GlcNAc-modified proteins after stripping. Red boxes are bands showed in the main text. X means samples were not used in data analysis.

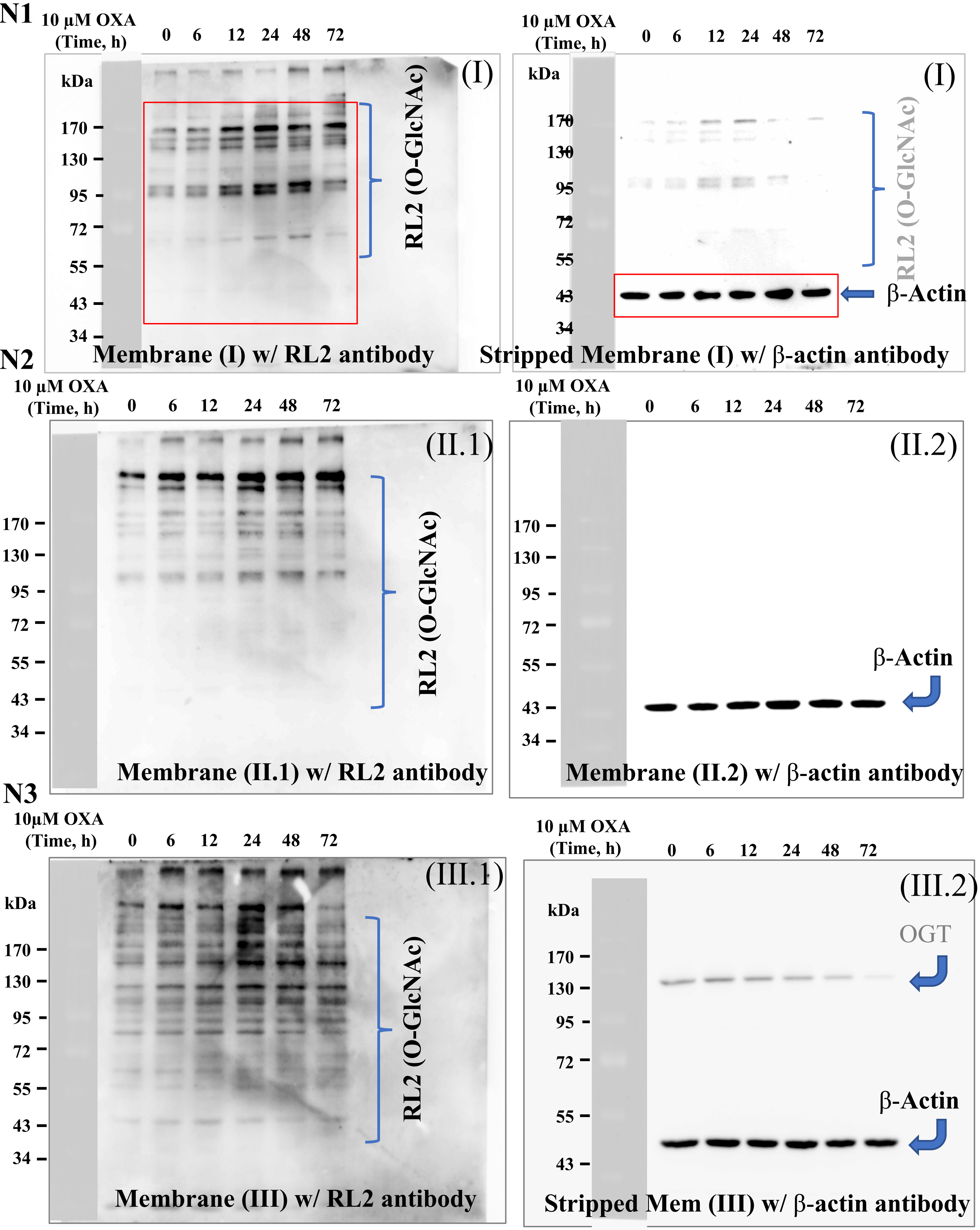

Figure S9. Raw data shown in Figure 1C. *O*-GlcNAcylation level of SW620 cells treated with OXA (0-72 hours, at 10 µM). Immunoblots (IB) of *O*-GlcNAc modified proteins (RL2) and β-actin on Membrane (I, II, and III) of 3 independent replicates, respectively. Membranes were firstly probed by RL2 antibody, stripped and re-probed with β-actin antibody as indicated in the figures. Gray texts represent the remaining band intensities of *O*-GlcNAc-modified proteins/OGT after stripping as indicated. Red boxes are bands showed in the main text.

Combination treatments 50 µM OSMI-1 + 10 µM OXA (N1)

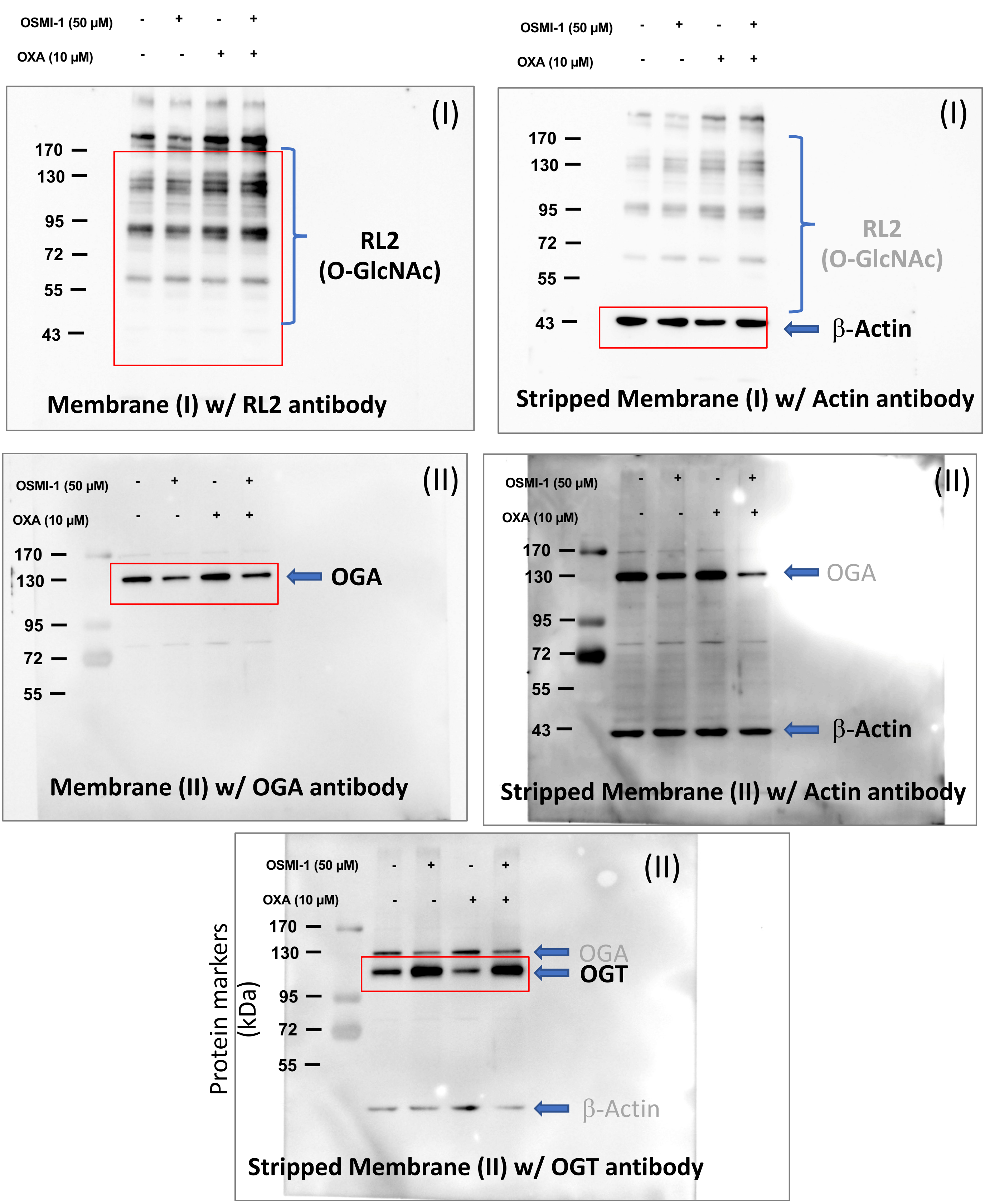

Figure S10. Raw data shown in Figure 2A. The levels of *O*-GlcNAcylation, OGT, OGT, and β-actin of SW620 cells treated with 10 µM OXA and 50 µM OSMI-1 (N1 of 3 independent replicates). Immunoblots (IB) of *O*-GlcNAc modified proteins (RL2) and β-actin on Membrane (I) and OGA, β-actin, and OGT on Membrane II, respectively. Membranes were probed by antibodies as indicated in the figures, stripped and re-probed with later antibodies as indicated in the figures. Gray texts represent the remaining band intensities of indicated proteins after stripping. Red boxes are bands showed in the main text.

Combination treatments 50 μM OSMI-1 + 10 μM OXA (N2 and N3)

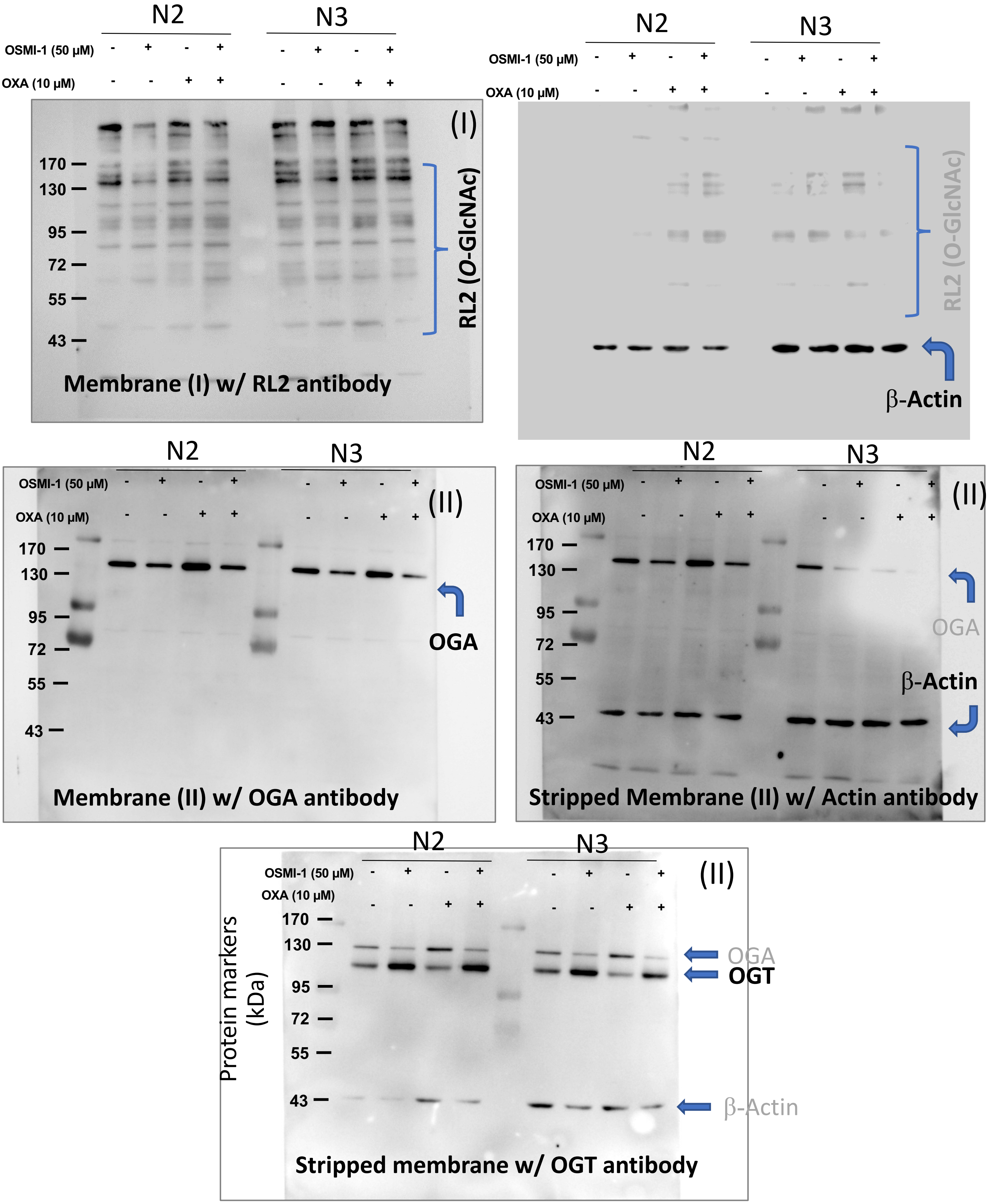

Figure S11. Raw data shown in Figure 2A. The levels of *O*-GlcNAcylation, OGT, OGT, and β-actin of SW620 cells treated with 10 μM OXA and 50 μM OSMI-1 (N2 and N3 of 3 independent replicates)**.** Immunoblots (IB) of O-GlcNAc modified proteins (RL2) and β-actin on Membrane (I) and OGA, β-actin, and OGT on Membrane II, respectively. Membranes were probed by antibodies as indicated in the figures, stripped, and re-probed with later antibodies as indicated in the figures. Gray texts represent the remaining band intensities of indicated proteins after stripping.

Combination treatments OGT knockdown + 10 μM OXA, 48 h (N1 and N2)

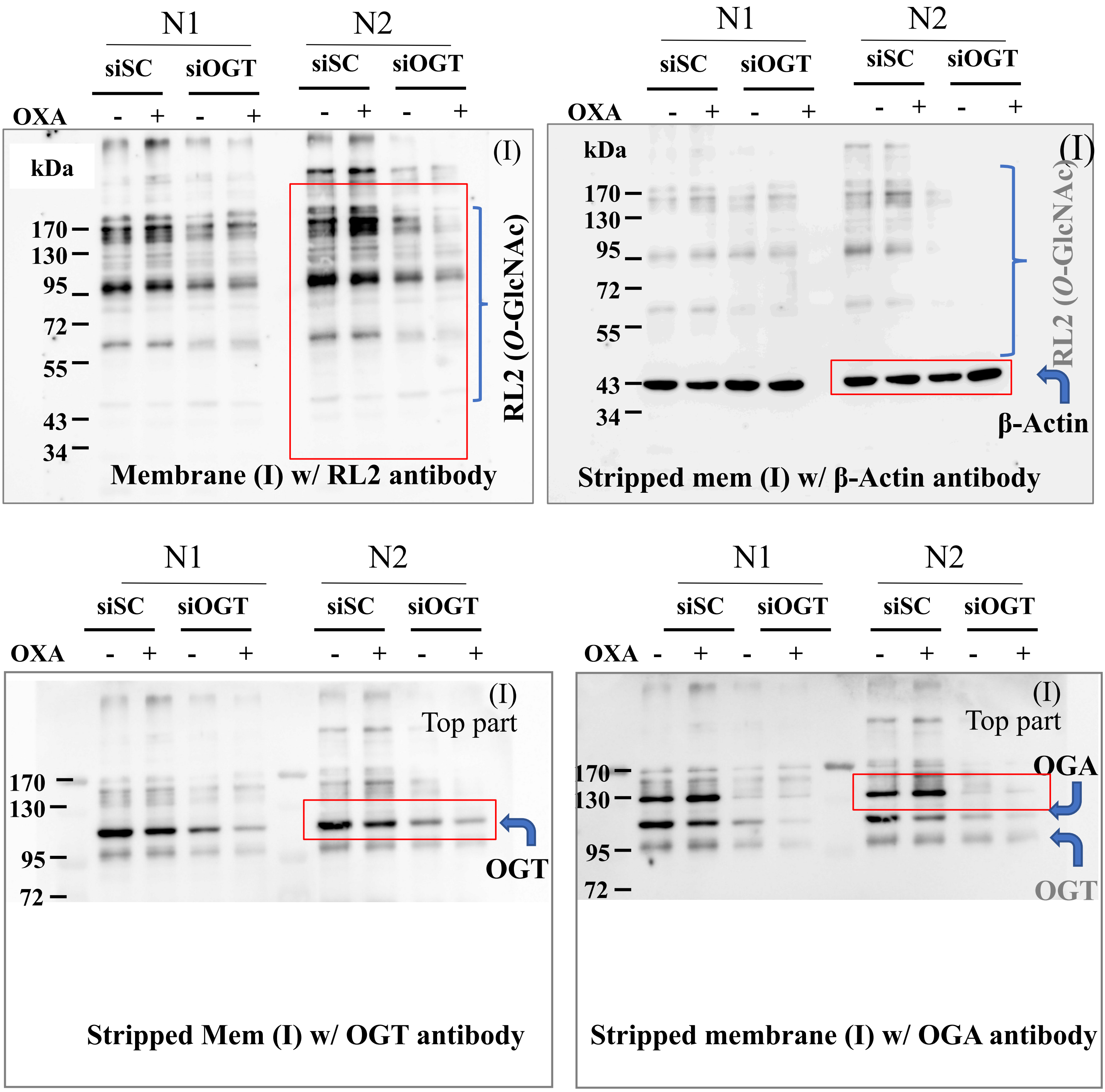

Figure S12. Raw data shown in Figure 3A. The levels of *O*-GlcNAc, OGA, OGT and β-actin of SW620 CRC cells treated with 10 μM OXA and OGT knockdown (N1 and N2 of 3 independent replicaates). Immunoblots (IB) of *O*-GlcNAc modified proteins (RL2) and β-actin, OGA, β-actin, and OGT on Membrane (I). Membrane (I) was probed firstly with *O*-GlcNAc RL2 antibody, then stripped, and re-probed with later antibodies as indicated in the figures. Gray texts represent the remaining band intensities of indicated proteins after stripping. Red boxes are bands showed in the main text.

Combination treatments OGT knockdown + 10 µM OXA, 48 h (N3)

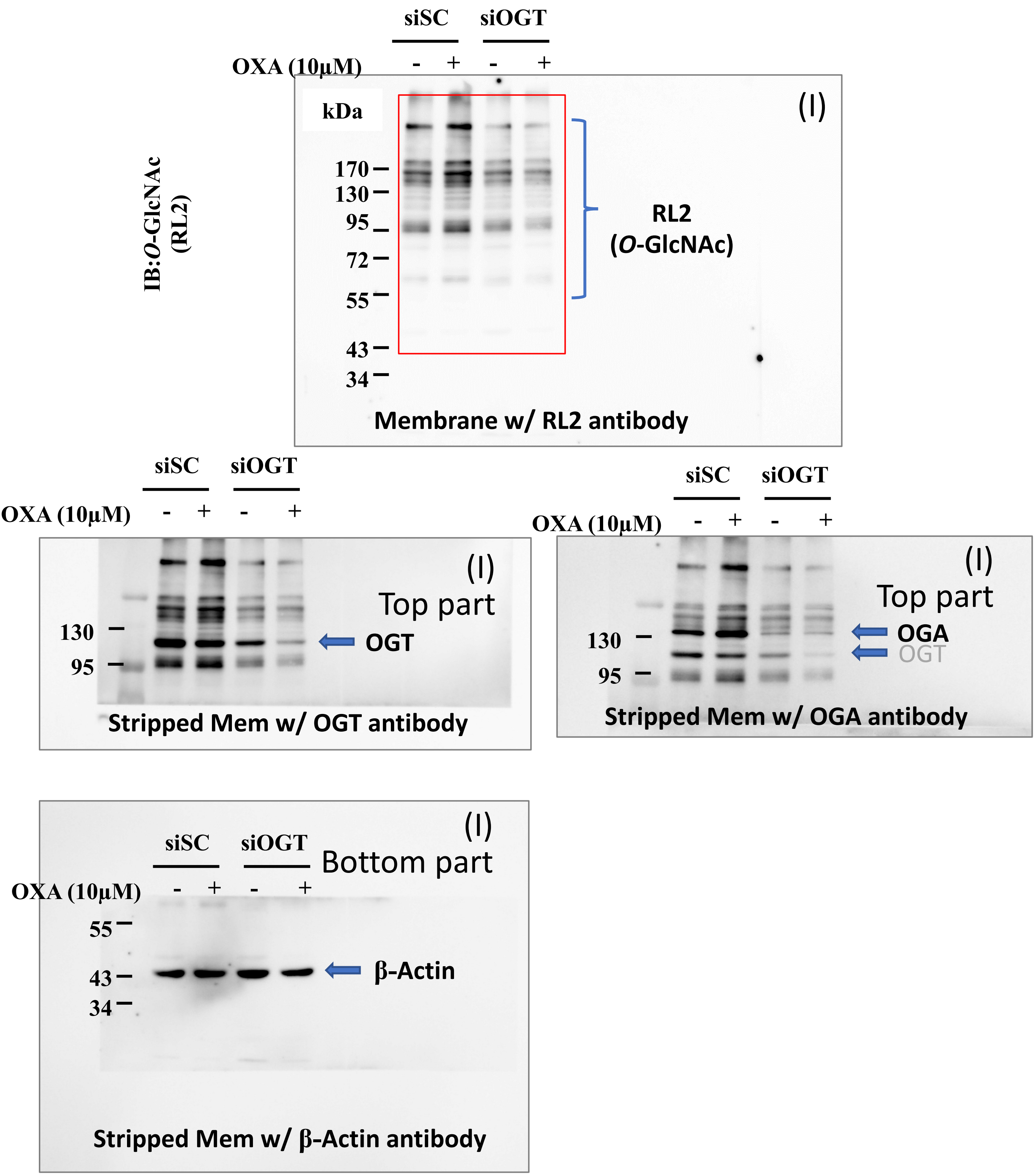

Figure S13. Raw data shown in Figure 3A. The levels of *O*-GlcNAc, OGA, OGT and β-actin of SW620 CRC cells treated with 10 µM OXA and OGT knockdown (N3 of 3 independent replicates). Immunoblots (IB) of *O*-GlcNAc modified proteins (RL2) and β-actin, OGA, β-actin, and OGT on Membrane (I). Membrane (I) was probed firstly with *O*-GlcNAc RL2 antibody, then stripped, and re-probed with later antibodies as indicated in the figures. Gray texts represent the remaining band intensities of indicated proteins after stripping. Red box is bands showed in the main text.

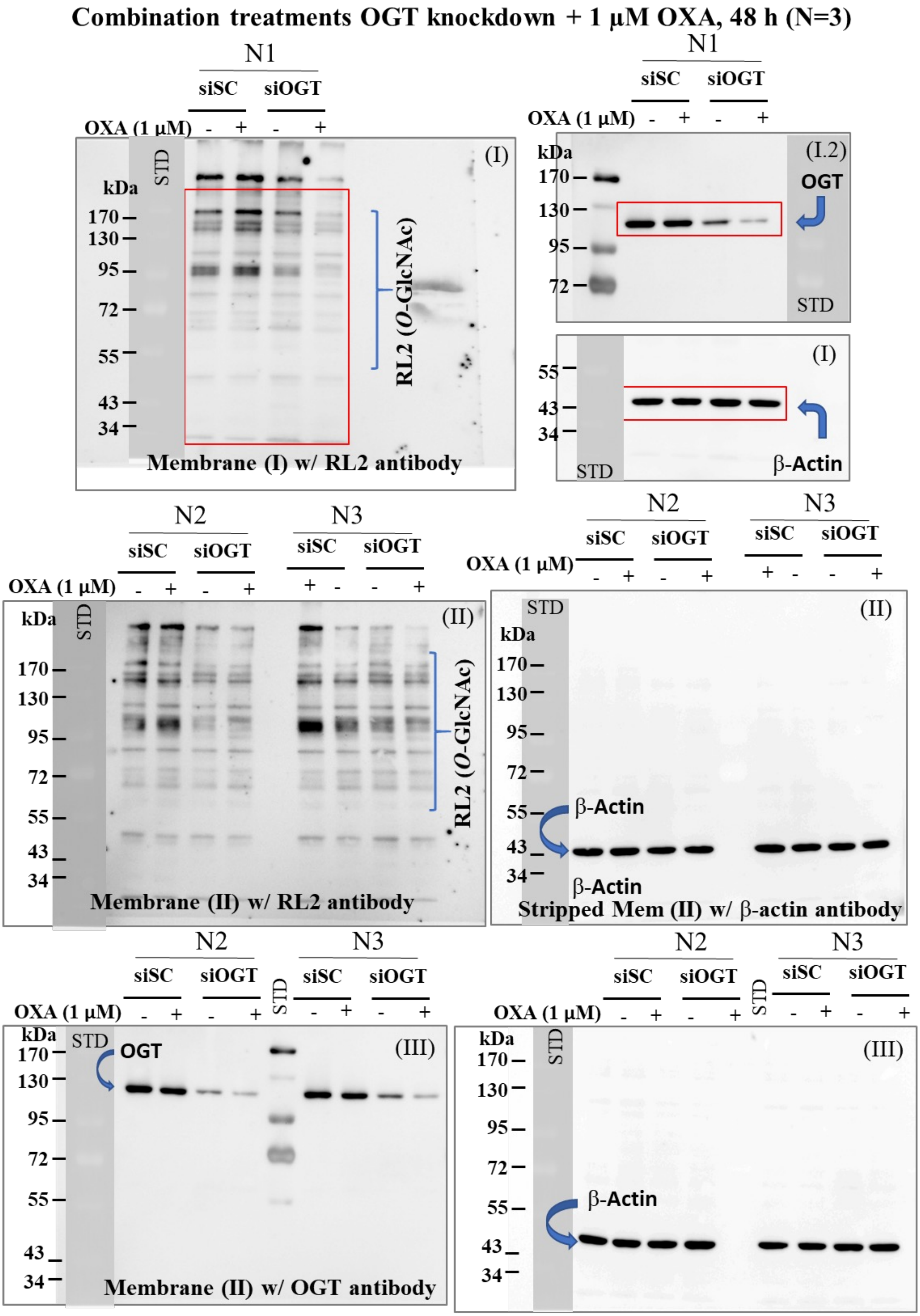

Figure S14. Raw data shown in Fig S3. The levels of *O*-GlcNAc, OGA, OGT and β-actin of SW620 CRC cells treated with 1 µM OXA and OGT knockdown (3 independent replicates). Immunoblots (IB) of *O*-GlcNAc modified proteins (RL2) and β-actin, OGA, β-actin, and OGT on Membranes. Membranes were probed firstly with *O*-GlcNAc RL2 antibody, then stripped, and re-probed with later antibodies as indicated in the figures. Red boxes are bands showed in the supplementary data.

Combination treatments 50 µM OSMI-1 + 10 µM OXA (N1, N2 and N3)

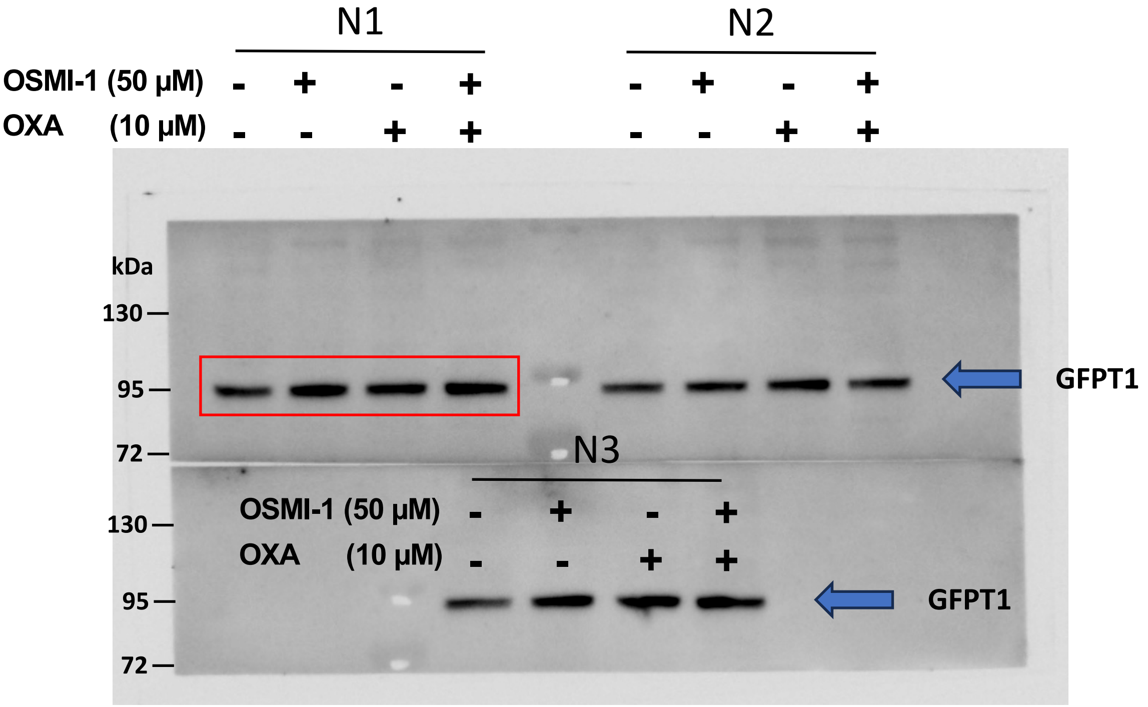

Figure S15. Raw data shown in Figure 2A. The level of GFPT1 of SW620 cells treated with 10 µM OXA and 50 µM OSMI-1 (3 independent replicates). Immunoblots (IB) of GFPT1 on Membranes. Membranes were probed by GFPT1 antibody. Red box is bands showed in the main text.

Combination treatments OGT knockdown + 10 µM OXA, 48 h (N1, N2 and N3)

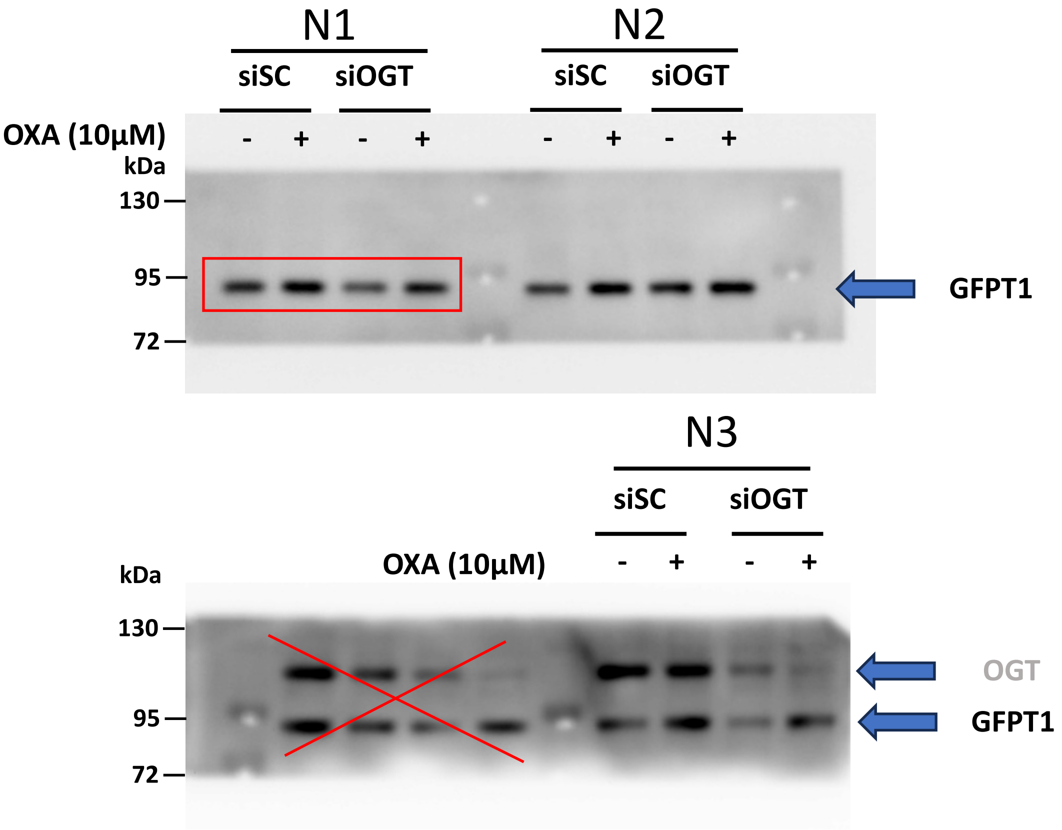

Figure S16. Raw data shown in Figure 3A and S6. The level of GFPT1 of SW620 CRC cells treated with 10 µM OXA and OGT knockdown (3 independent replicates). Immunoblots (IB) of GFPT1 on Membranes. Membranes (N1 and N2) were probed by GFPT1 antibody. Membrane (N3) was probed firstly with OGT antibody, then stripped, and re-probed with GFPT1 antibody. Gray texts represent the remaining band intensities of indicated proteins after stripping. Red box is bands showed in the main text.

Validation of proteins in signaling pathways affected by OXA and OGT knockdown

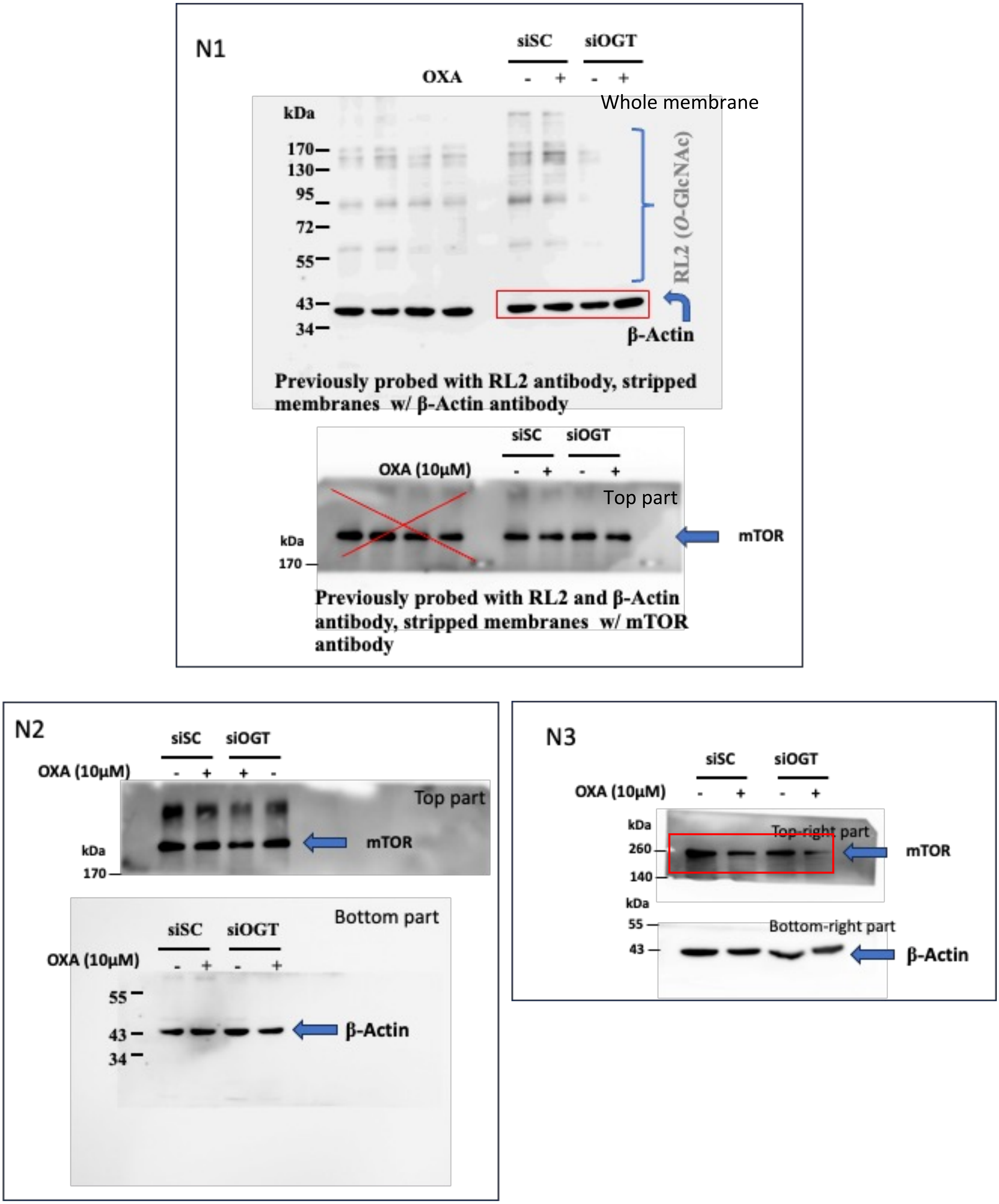

Figure S17. Raw data shown in Figure S6. The levels of mTOR and β-actin of SW620 CRC cells treated with 10 μM OXA and OGT knockdown (3 independent replicates). Immunoblots (IB) of mTOR and β-actin on Membranes. Membranes (N1, N2 and N3) were probed by mTOR and β-actin antibodies. Red box is bands showed in the main text.

Validation of proteins in signaling pathways affected by OXA and OGT knockdown

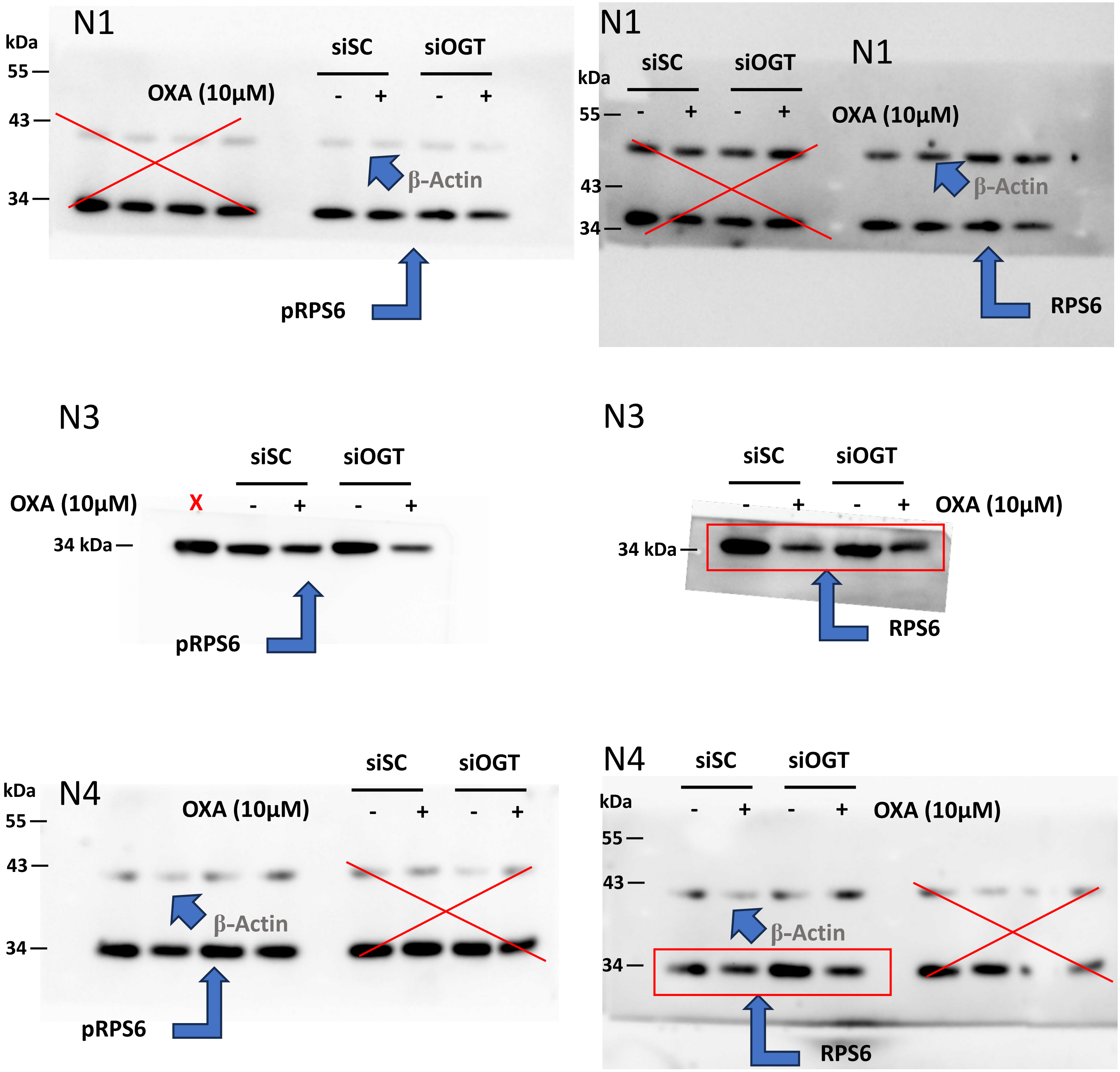

Figure S17. Raw data shown in Figure 7A. The levels of pRPS6 and RPS6 of SW620 CRC cells treated with 10 μM OXA and OGT knockdown (3 independent replicates). Immunoblots (IB) of pRPS6 and RPS6 on Membranes. Membranes (N1, N3 and N4) were probed by pRPS6 and RPS6 antibodies. Red boxes are bands showed in the main text.
